# Synthesis of Substituted Acyclic and Cyclic *N*‐Alkylhydrazines by Enzymatic Reductive Hydrazinations

**DOI:** 10.1002/cbic.202400700

**Published:** 2024-10-29

**Authors:** Niels Borlinghaus, Donato Calabrese, Lars Lauterbach, Bettina M. Nestl

**Affiliations:** ^1^ Institute of Biochemistry and Technical Biochemistry Department of Technical Biochemistry Universitaet Stuttgart Allmandring 31 70569 Stuttgart Germany; ^2^ Institute of Applied Microbiology (iAMB) RWTH Aachen University Worringer weg 1 52074 Aachen Germany; ^3^ Innophore GmbH Am Eisernen Tor 3 8010 Graz Austria

**Keywords:** Biocatalysis, Bioorganic chemistry, Cofactor regeneration, Hydrazines, *N*-Heterocycles

## Abstract

Imine reductases (IREDs) provide promising opportunities for the synthesis of various chiral amines. Initially, asymmetric imine reduction was reported, followed by reductive aminations of aldehydes and ketones *via* imines. Herein we present the reductive amination of structurally diverse carbonyls and dicarbonyls with hydrazines (reductive hydrazination), catalyzed by the IRED from *Myxococcus stipitatus*. In analogy to IRED‐catalyzed reductive aminations, various carbonyls and dicarbonyls could react with simple hydrazines to produce substituted acyclic and cyclic *N*‐alkylhydrazines. By incorporating and scaling up a hydrogenase cofactor regeneration system, we demonstrated the scalability and atom‐efficiency of an H_2_‐driven double reductive hydrazination, highlightling the potential of IREDs in biocatalysis.

## Graphical abstract

Imine reductases (IREDs) offer promising synthesis routes for chiral amines. We have demonstrated reductive amination of diverse carbonyls and dicarbonyls with hydrazines, catalyzed by *Myxococcus stipitatus* IRED. Incorporating a hydrogenase cofactor regeneration system showcases the scalability and atom‐efficiency of H_2_‐driven double reductive hydrazination, highlighting IREDs’ biocatalytic potential.

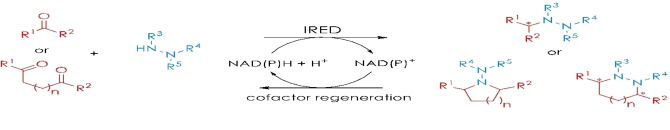

Reductive aminations are among the most convenient and straightforward reactions for amine synthesis – essential building blocks for the development of agrochemicals and pharmaceutical agents.^[1]^ In the recent years, NADPH‐dependent imine reductases (IREDs) have emerged as efficient catalysts for reductive aminations, enabling the use of a range of amines – spanning from primary and secondary alkyl amines, aryl amines, hydroxyl amines, diamines, piperidines to pyrrolidines.[^2^, ^3^, ^4^, ^5^, ^6^, ^7^, ^8^] Hydrazines are widely used in the pharmaceutical, agricultural and rocket fuel industries and are building blocks for *N*‐heterocyclic compounds such as pyrazoles, pyrazines and indoles.[^9^, ^10^] The presence of hydrazine as an intermediate in nitrogenases illuminates the diverse roles of hydrazine compounds in biological systems.^[11]^ To date, however, there have been limited biocatalytic methods reported in the literature that could implement reductive aminations using hydrazines as reactants, so‐called reductive hydrazinations.^[12]^ This is intriguing, given the numerous biologically active hydrazine products found in nature with anti‐microbial, anti‐fungal, anti‐oxidative or anti‐cancerogenic properties.[^13^, ^14^, ^15^] Most of these alkaloids are derived from simple hydrazine precursors like phenylhydrazine, 4‐hydroxymethyl phenylhydrazine, *N*‐aminoalanine, or piperazic acid.[^12^, ^13^] Interestingly, there are biological studies indicating the occurrence of methylhydrazine in the fungus *Gyromitra esculenta* and *N*,*N*‐dimethylhydrazine in tobacco, which could act as a substrate for the formation of more complex hydrazines, hydrazidines or hydrazones.[^16^, ^17^]

Based on our recent results, earlier experiences, and in continuation of our research activities, we anticipated the use of IREDs could enable an efficient reductive hydrazination process. Mirroring the requirements for reductive amination (Scheme 1A), a method for reductive hydrazination should facilitate the condensation of a carbonyl with one of the vicinal amino groups of hydrazine to generate a hydrazone.[^18^, ^19^] This intermediate must then be selectively reduced over the carbonyl starting material. In addition to reductive hydrazinations, we envisioned that hexahydropyridazines, 1,2 diazepanes, *N*‐aminopyrrolidines and *N*‐aminopiperidines could be obtained by utilizing dicarbonyls with hydrazines in double reductive hydrazination reactions (Scheme 1B).

**Scheme 1:**
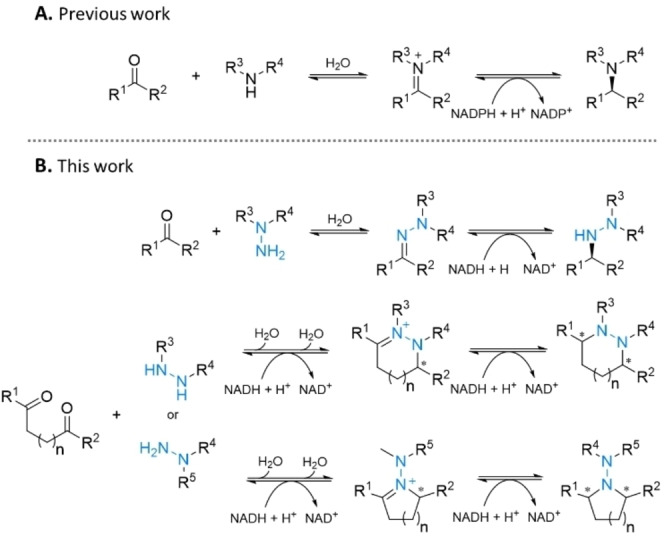
Imine reductase‐catalyzed reductive amination (A) *versus* presented reductive hydrazinations (B).

The research in our laboratories has thus focused on reductive hydrazinations using IREDs for the facile preparation of hydrazines in one‐pot under mild conditions.

We used an engineered NADH‐dependent IRED variant and found that it was effective in the application of our method. We thus also address the scalability of this biocatalytic process, acknowledging the challenges posed by the stoichiometric supply for nicotinamide cofactors during upscaling. Building on atom‐efficient integration of the soluble [NiFe]‐hydrogenase (SH) from *Cupriavidus necator* with IREDs for H_2_‐driven reductive amination, we extended this approach to double reductive hydrazination.[^20^, ^21^, ^22^]

In an earlier study, we have reported on a promising method for producing piperazines and azepanes enzymatically through double reductive aminations. To conduct these reactions, we utilized the *R*‐selective IRED from *Myxococcus stipitatus* (*R*‐IRED_*Ms*).^[23]^ We further engineered the cofactor specificity of *R*‐IRED_*Ms* from NADPH to NADH yielding in variant *R*‐IRED_*Ms*‐V8. After demonstrating that variant V8 shows similar catalytic performance compared to the wild‐type enzyme (data not shown), we decided to use this variant for probing the reaction of various aldehydes and ketones in the presence of hydrazines in a spectrophotometric NADH‐depletion assay. We employed seven different hydrazines (a–g) and 29 aldehydes and ketones (1–29) to inspect the substrate scope and limitations of the enzymatic reductive hydrazination. The results are detailed in Tables 1 and 2.

**Table 1:** Substrate scope of enzymatic reductive hydrazinations of linear aldehydes and ketones.^[a]^ The table lists all substrates used as starting materials, but only products with a turnover frequency (TOF)>1 min^−1^ are shown as entries. Details of the remaining reactions can be found in the Supporting Information.

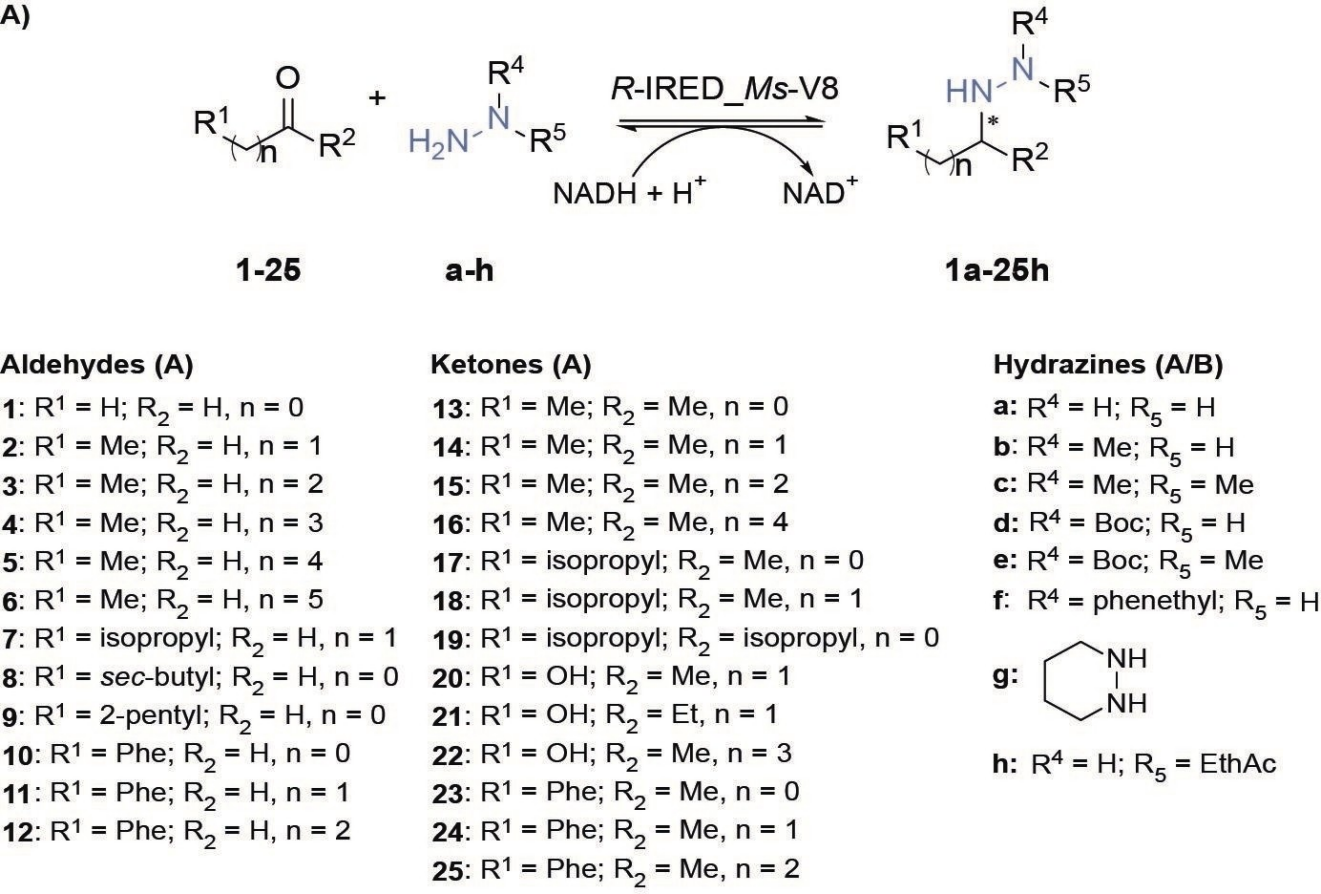		
Entry	Hydrazine product	TOF (min^−1^)
1	**2 b**	6.9±0.4
2	**3 a**	4.6±0.2
3	**3 b**	9.0±0.3
4	**3 c**	1.3±0.1
5	**4 b**	6.3±0.6
6	**9 b**	1.2±0.1
7	**14 b**	1.3±0.2

[a] Reactions performed with 5 mM of carbonyl starting material, 5 mM hydrazine (1 eq), 2.5 mM NADH cofactor, 15 μM *R*‐IRED_*Ms*‐V8 (≙0.5 mg mL^−1^), 30 mM glucose‐6‐phosphate, 2.5 mM MgCl_2_, 5 U mL^−1^ glucose‐6‐phosphate dehydrogenase in 100 mM phosphate buffer pH 6.0 at 25 °C for 4 h.

**Table 2:** Substrate scope of enzymatic reductive hydrazinations of cyclic ketones.^[a]^

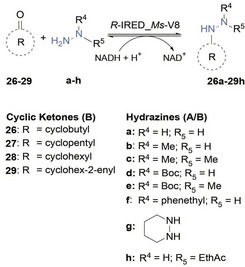		
entry	hydrazine product	TOF (min^−1^)
1	**26 b**	0.31±0.04
2	**27 b**	0.57±0.08
3	**28 a**	29±3
4	**28 b**	36±2
5	**28 c**	2.6±2
6	**28 d**	0.06±0.01
7	**28 e**	0.04±0.01
8	**29 b**	0.16±0.05

[a] Reactions performed with 5 mM of carbonyl starting material, 5 mM hydrazine (1 eq), 2.5 mM NADH cofactor, 15 μM *R*‐IRED_*Ms*‐V8 (≙ 0.5 mg mL^−1^), 30 mM glucose‐6‐phosphate, 2.5 mM MgCl_2_, 5 U mL^−1^ glucose‐6‐phosphate dehydrogenase in 100 mM phosphate buffer pH 6.0 at 25 °C for 4 h.

We found that *R*‐IRED_*Ms*‐V8 is an effective catalyst as most enzymatic reactions provide the desired hydrazine products. The reductive hydrazination rate of aldehydes and ketones to hydrazines on the catalyst are expressed as turnover frequencies (TOF) as a measure of activity obtained via the spectrophotometric assay. The TOF indicates how many times the catalytic cycle occurs (turns) on a single site per unit time and is typically defined as the number of reaction products generated per active site per unit time. We chose to describe the enzyme activity using TOF, when a spectrophotometric assay was possible, to provide a clear indication of the activity at the single catalyst active site level. We confirmed the formation of these products through biotransformations. Due to the absence of hydrazine standards, the formed products were identified using GC‐MS (SI Chapter 4). We observed that *R*‐IRED_*Ms*‐V8 accepted various carbonyl substrates, and in the absence of IRED catalyst, no product formation was observed and starting materials remained unaffected or reacted to the hydrazone intermediate without further reduction reactions. In general, higher activities were observed for aliphatic aldehydes 1–9 (Table 1, entries 1–6) compared to (non‐cyclic) aliphatic ketones 13–22 (Table 1, entry 7). Thereby, the best accepted non‐cyclic carbonyls were the aliphatic C4‐substrates *n*‐butanal (3, Table 1, entries 2–4, best aldehyde) and 2‐butanone (14, Table 1, entry 7, best non‐cyclic ketone). Moreover, cyclohexanone (28, Table 2, entries 3–7) was found to afford the highest turnover frequency in the reductive hydrazination.

More bulky hydrazines and hydrazines protected with a BOC group were also compatible with phenylacetone (24, SI Table S2, entries 11–13) and 28. The comparison of the hydrazine substrates tested with carbonyl substrates **3 a**–**3 g** (Table 1, entries 3–4), **24 a**–**24 g** (SI Table S2, entries 11–13) and **28 a**–**28 g** (Table 2, entries 3–7), revealed that methylhydrazine (b) is best accepted by IRED. Hydrazine esters were also tested, such as ethyl hydrazinoacetate (h), but no activity has been detected.

Moreover, for some carbonyl and hydrazine combinations no activity could be detected in the spectrophotometric assay. Some hydrazone intermediates **11 a**, **11 b**, **12 b**, **24 d** caused the formation of a turbid solution or misleading signals in the NADH‐depletion assay (**3 g**, **24 g**, **28 g**). Despite these results, we were able to confirm the product formation by GC‐MS for most of these substrate combinations (SI Chapter 4).

It is noteworthy that some of the hydrazine products formed can act as nucleophiles for subsequent reductive hydrazinations with carbonyl substrates (Scheme S1). However, these secondary products were detected only in traces and can be neglected for most reactions. The exception to this is the transformation of 3 or 28 with hydrazine (a). In this substrate combination, secondary reactions were significantly increased, but we were able to circumvent this issue by increasing the hydrazine concentration and shortening the reaction time (Figure S1). Next, we continued to examine the reaction scope with various dicarbonyls to enable double reductive hydrazination for the formation of cyclic hydrazines. For this reaction, two consecutive IRED‐catalyzed steps along with the consumption of two nicotinamide molecules are necessary to produce saturated *N*‐heterocycles (Scheme S2).

In principle, there are two approaches how the double reductive hydrazination of dicarbonyls can proceed, depending on the substitution pattern of the hydrazine substrate – either both (subtype 1) or one vicinal amino group is incorporated into cyclic pyrazolidine or hexahydropyridazine products (subtype 2). For some hydrazine substrates, both subtypes are possible. In total, nine different saturated heterocycles were formed applying the double reductive hydrazination. The results are summarized in Table 3.

**Table 3:** Product formations in IRED‐catalyzed double reductive hydrazination reactions.^[a]^

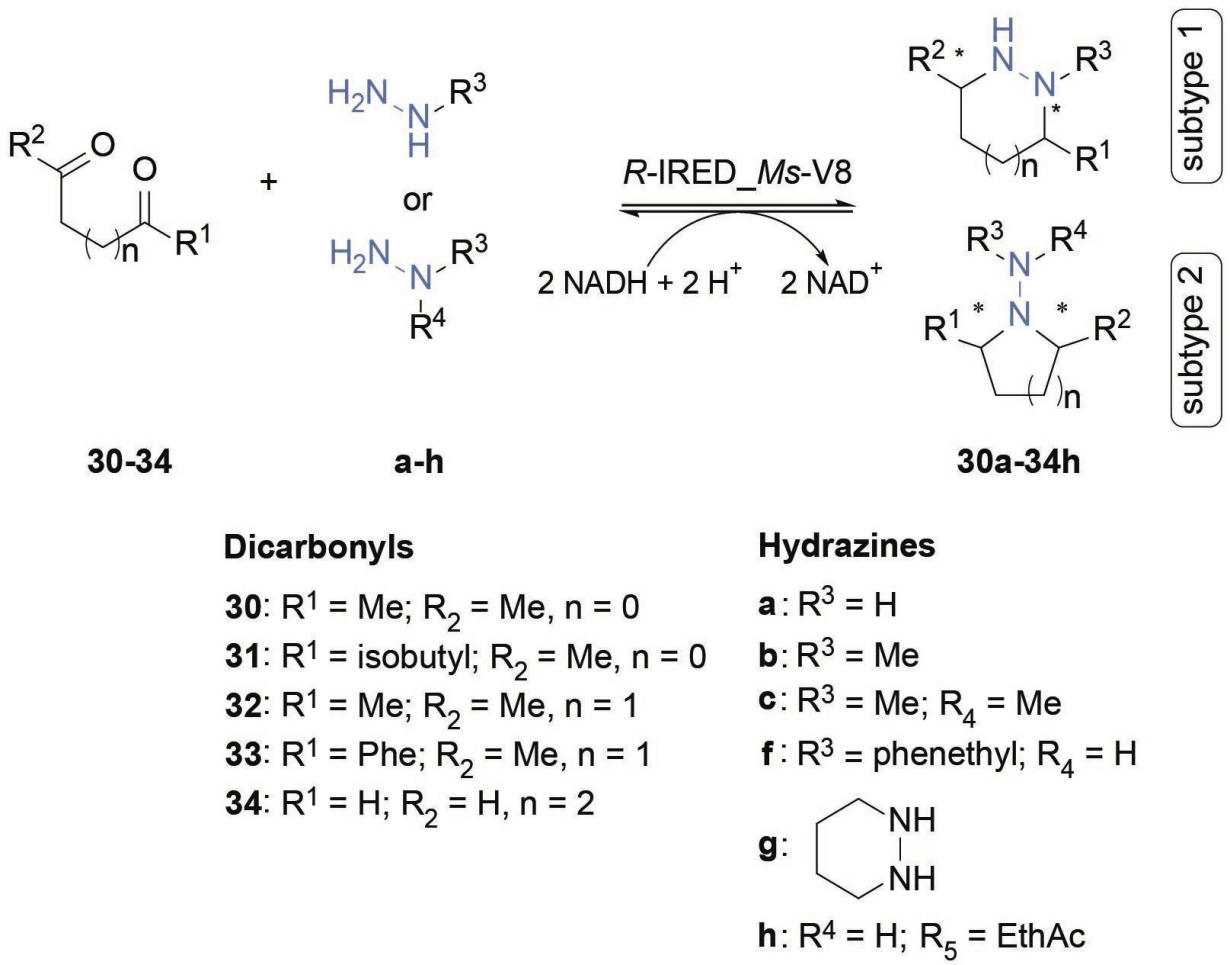		
entry	hydrazine product	Product formation (%)^[b]^
1	**30 b**	–
2	**31 b**	–
3	**32 a**	6±1
4	**32 b**	14±1
5	**32 c**	4±0.2
6	**32 f**	–
7	**33 a**	<1
8	**33 b**	–
9	**34 a**	<1
10	**34 b**	9±1
11	**34 c**	94±4
12	**34 f**	<1
13	**34 g**	10±1

[a] Reactions performed with 5 mM of carbonyl starting material, 5 mM hydrazine (1 eq), 2.5 mM NADH cofactor, 15 μM *R*‐IRED_*Ms*‐V8 (≙0.5 mg Ml^−1^), 30 mM glucose‐6‐phosphate, 2.5 mM MgCl_2_, 5 U mL^−1^ glucose‐6‐phosphate dehydrogenase in 100 mM phosphate buffer pH 6.0 at 25 °C for 4 h. [b] Products were identified *via* GC‐MS and product formation was calculated based on the GC‐FID‐signal. For the calibration, we used acetylated hexahydropyridazine.

Results obtained from GC‐MS showed that in some cases, there was an accumulation of the cyclic hydrazone intermediate, formed after the first IRED‐catalyzed reduction step. Based on these results, we can conclude that for certain substrate combinations, the second reduction step by the IRED is the rate limiting step. As a result, it is not possible to determine the overall activity of the total reaction using the NADH‐depletion assay. We thus characterized the enzymatic activity measuring endpoint yields via GC‐MS and GC‐FID.

Since there were no product standards available, we used diacetylated hexahydropyridazine **34 g** (Table 3, entry 13) for calibration curves and calculations of product formations (Table 3). No product formation was observed with diketones 30 and 31 (Table 3, entries 1–2), as they rapidly reacted with hydrazines to form a pyrazole side product, as described in literature.^[24]^ Interestingly, the highest product formation of 94 % was observed with glutaraldehyde (34, Table 3, entries 9–13) and 1,1‐dimethylhydrazine (c), which only allows the reaction *via* subtype 2. Moderate product formations of up to 14 % were detected for substrates 32 and 33 (Table 3, entries 3–13). Remarkably, despite its high toxicity to proteins, 34 was found to be a suitable dicarbonyl for the double reductive hydrazination. Incubation of 34 with the hydrazine substrate for 30 min before adding IRED resulted in a reduced concentration of toxic 34 due to the formation of the less toxic hydrazone intermediate, which is then converted into the product in followed enzymatic reductions. Overall, our studies have shown that hydrazines are accepted nucleophiles for the enzymatic reductive hydrazination and can be used to form acyclic hydrazine products as well as hydrazine‐containing *N*‐heterocycles with different substitution patterns. Turnover frequencies of up to 36 min^−1^ (≙1.1 U mg^−1^) were reached for the reductive hydrazination of carbonyls. The highest product formation (94 %) was observed for the double reductive hydrazination of 34 with c to generate *N,N*‐dimethylpiperidin‐1‐amine (**34 c**, Table 3, entry 11). Notably, although chiral centers are formed during the double reductive hydrazination, the lack of chiral standards prevented us from fully discriminating the enantiomeric excess in the products.

While the initial biocatalytic system utilized GDH for cofactor regeneration, we aimed to improve the efficiency by integrating the SH with *R*‐IRED_*Ms*‐V8, thereby establishing an H_2_‐driven double reductive hydrazination process. We chose compound **34 c** as a proof‐of‐concept, which we prepared by converting glutaraldehyde (34) and 1,1‐dimethylhydrazine (c) using the IRED. In this setup, we increased the reaction volumes to mimic a technical scale and adjusted the cofactor concentration, to maximize efficiency. We also extended the reaction time to eight hours to account for changes in reaction kinetics due to lower component concentrations. These changes were crucial in maintaining high conversion rates without sacrificing efficiency. We were able to achieve a 92 % conversion rate under these conditions, as evidenced by the results in Table 4.

**Table 4:** H_2_‐driven biocatalytic double reductive hydrazination.^[a]^

	Analytical Scale (Glucose/GDH)	Upscaling (H_2_/SH)
Volume [mL]	0.25	10
Yield [%]	94±4	92±6
Cofactor [mM]	2.5	0.4
SH TTN*	–	1860
*R*‐IRED_*Ms*‐V8 TTN*	626	932
Cofactor TTN**	3.92	23.3

[a] The reactions were conducted using 5 mM of dicarbonyl (34) and 5 mM of hydrazine (c), complemented by 0.4 mM NAD^+^, 15 μM *R*‐IRED_*Ms*‐V8, 2.5 μM SH. The system was buffered in an H_2_ saturated 100 mM phosphate buffer at pH 7.0. The reaction were carried out at 25 °C over a period of 8 h. *TTN was calculated as (n_product_/n_enzyme_). **TTN was calculated as (n_product_/n_NAD+_)

To accurately describe the stereoselectivity of biocatalytic reductive hydrazination, the development and use of chiral standard materials, higher catalytic activities for product isolation, and advanced chiral analytical methods are required. The catalyst *R*‐IRED_*Ms*‐V8, noted for its effectiveness in the asymmetric synthesis of chiral amines, is anticipated to exhibit similar selectivity in reductive hydrazination. Preliminary chiral chromatographic data supports this hypothesis, showing enantiomeric excess (*ee*) greater than 75 % for highly active catalyst‐substrate combinations (data not shown).

In summary we have successfully developed an enzymatic approach for the synthesis of hydrazine derivatives through IRED‐catalyzed reductive hydrazinations of (di)carbonyls. To achieve this, we used our previously engineered NADH‐dependent IRED variant (*R*‐IRED_*Ms*‐V8). With this engineered catalyst, we were able to form non‐cyclic hydrazines and prepare novel *N*‐heterocycles, resulting in the production of pyrazolidine, hexahydropyridazine, pyrrolidine‐1‐amine or piperidine‐1‐amine products. We addressed the challenges of cofactor regeneration by integrating the soluble [NiFe]‐hydrogenase from *Cupriavidus necator*, enhancing atom efficiency and sustainability for large‐scale applications. By developing an H_2_‐driven double reductive hydrazination biocatalytic process, we have not only provided a mild and straightforward method for developing novel *N*‐heterocyclic compounds but also set a new benchmark for sustainable green synthesis of hydrazine derivatives. Further studies on the synthetic applications and improvement of IRED catalysts are ongoing in our group.

## Author Contributions

D.C. and N.B. performed all experiments, analyzed the results, and wrote the manuscript. B.M.N and L.L. developed the original concept and guided the outline of the study. All authors contributed to writing and evaluating the manuscript.

## Conflict of Interests

The authors declare no conflict of interest.

## Supporting information

As a service to our authors and readers, this journal provides supporting information supplied by the authors. Such materials are peer reviewed and may be re‐organized for online delivery, but are not copy‐edited or typeset. Technical support issues arising from supporting information (other than missing files) should be addressed to the authors.

Supporting Information

## Data Availability

The data that support the findings of this study are available in the supplementary material of this article.
