# Supplementary material for: Synthesis of Substituted Acyclic and Cyclic N‐Alkylhydrazines by Enzymatic Reductive Hydrazinations
Source: Chembiochem. 2024 Oct 29;26(4):e202400700. doi: 10.1002/cbic.202400700 (PMC11833756; doi:10.1002/cbic.202400700)
Supplement: Supplementary file 1 — Supporting Information [file CBIC-26-e202400700-s001.pdf]

ChemBioChem

Supporting Information

# Synthesis of Substituted Acyclic and Cyclic *N*-Alkylhydrazines by Enzymatic Reductive Hydrazinations

Niels Borlinghaus, Donato Calabrese, Lars Lauterbach,* and Bettina M. Nestl*

# Synthesis of Substituted Acyclic and Cyclic *N*-Alkylhydrazines by Enzymatic Reductive Hydrazinations

## Supporting information

Niels Borlinghaus[1], Donato Calabrese[2], Lars Lauterbach[2]* and Bettina M. Nestl[1][3]*

[1] Institute of Biochemistry and Technical Biochemistry, Department of Technical Biochemistry, Universitaet Stuttgart, Allmandring 31, 70569 Stuttgart, Germany.

[2] Institute of Applied Microbiology (iAMB), RWTH Aachen University, Worringer weg 1, 52074 Aachen, Germany.

[3] Innophore GmbH, Am Eisernen Tor 3, 8010 Graz, Austria.

**Content**

1 Materials and methods ........ 1

2 Supporting figures and tables ........ 2

3 General procedures ........ 4

4 GC chromatograms ........ 6

5 References ........ 29

# 1 Materials and methods

**(A) Chemicals:** Except otherwise noted, all solvents, buffers components and chemicals were obtained from Sigma-Aldrich and Fluka (Steinheim, Germany), Carl Roth GmbH (Karlsruhe, Germany), Acros Organics (Geel, Belgium), VWR (Darmstadt, Germany), abcr GmbH (Karlsruhe, Germany) and Alfa Aesar (Karlsruhe, Gemany).

**(B) Enzymes:** Glucose-6-phosphate dehydrogenase from *Leuconostoc mesenteroides* was purchased from Alfa Aesar (Karlsruhe, Germany).

**(C) DNA-Sequence:**

***R*-IRED_*Ms*-V8-his$_6$**

```
ATGGGCAGCAGCCATCATCATCATCATCACAGCAGCGGCCTGGTGCCGCGCGGCAGCCAT
ATGAAACCGACCCTGACCGTTATTGGCGCTGGCCGTATGGGCTCCGCACTGATTAAAGCA
TTCCTGCAATCTGGCTACACGACCACGGTGTGGGAATACGAGAAAGCCCGTAGCGAACCG
CTGGCAAAACTGGGCGCACATCTGGCTGATACGGTGCGTGACGCCGTTAAACGCAGCGAT
ATTATCGTGGTTAATGTGATTGATTATGACGTGTCTGATCAGCTGCTGCGCCAAGACGAA
GTGACGCGTGAACTGCGCGGCAAACTGCTGGTTCAGCTGACCAGCGGTTCTCCGGCACTG
GCTCGTGAACAGGAAACGTGGGCGCGCCAACATGGCATTGATTATCTGGACGGTGCGATC
ATGGCCACCCCGGATTTTATTGGCCAGGCAGAATGCGCTCTGCTGTACAGTGGTTCCGCG
GCCCTGTTCGAAAAACACCGTGCTGTCCTGAATGTGCTGGGCGGTGCCACCAGCCATGTC
GGCGAAGATGTTGGTCATGCCTCAGCACTGGACAGCGCCCTGCTGTTTCAGATGTGGGGC
ACCCTGTTCGGTACGCTGCAAGCACTGGCTATTTCTCGCGCAGAAGGCATCCCGCTGGAA
AAAACCACGGCGTTTATCAAACTGACCGAACCGGTCACCCAGGGTGCCGTTGCAGATGTC
CTGACCCGTGTTCAGCAAAATCGCCTGACCGCAGACGCTCAGACGCTGGCAAGTCTGGAA
GCTCATAACGTGGCGTTCCAACACCTGCTGGCCCTGTGTGAAGAACGTAATATCCATCGC
GGTGTTGCGGATGCCATGTACTCCGTTATTCGTGAAGCGGTCAAAGCCGGCCACGGTAAA
GATGACTTTGCAATTCTGACCCGCTTCCTGAAATAA
```

**(D) GC-MS analysis:** Gas chromatography-mass spectrometry was carried out on an Agilent GC 7890A instrument coupled with an Agilent 5975 Series mass selective detector (MSD) and an additional flame ionization detector. An Agilent DB-5 column (30 m × 250 µm × 0.25 µm) was operated with hydrogen as carrier gas (30 cm s$^{-1}$). Injector temperature: 250°C. Split mode with a split ratio of 10. Detector temperature: 320°C. Electron ionization of the analyte with 70 eV acceleration voltage.
In this study we used three different temperature programs (Table S1).

**Table S1: Applied temperature programs (A-C) for the GC-MS analysis.**

Temperature program A:

| **Rate** [°C min$^{-1}$] | **Final temp.** [°C] | **Hold** [min] |
|---|---|---|
| - | 100 | 1 |
| 25 | 310 | 2.5 |

Temperature program B:

| **Rate** [°C min$^{-1}$] | **Final temp.** [°C] | **Hold** [min] |
|---|---|---|
| - | 50 | 1 |
| 30 | 140 | 0 |
| 50 | 310 | 2.5 |

Temperature program C:

| **Rate** [°C min$^{-1}$] | **Final temp.** [°C] | **Hold** [min] |
|---|---|---|
| - | 50 | 1 |
| 25 | 125 | 2 |

## 2 Supporting figures and tables

### (A) Formation of secondary products:

During the biotransformation of **3** and **28** with **a**, we noticed the formation of some by-products that can act as nucleophiles for a secondary reaction. The secondary product appeared in the reaction following Scheme S2. In principle, a tertiary and quaternary product are possible for this substrates, but these could not be observed.

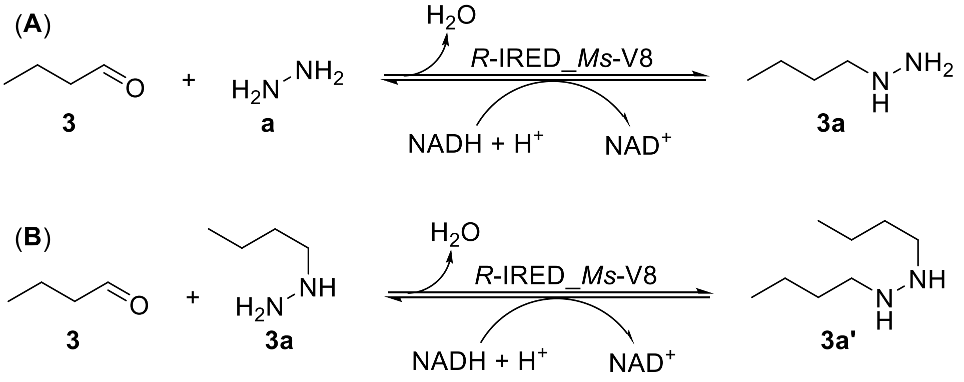


**Scheme S1:** Primary (A) and secondary (B) reactions during the biotransformation of **3** and **a** to **3a**.

By changing the substrate ratio and the reaction time, we were able to increase or decrease the formation of the secondary product (Figure S1).

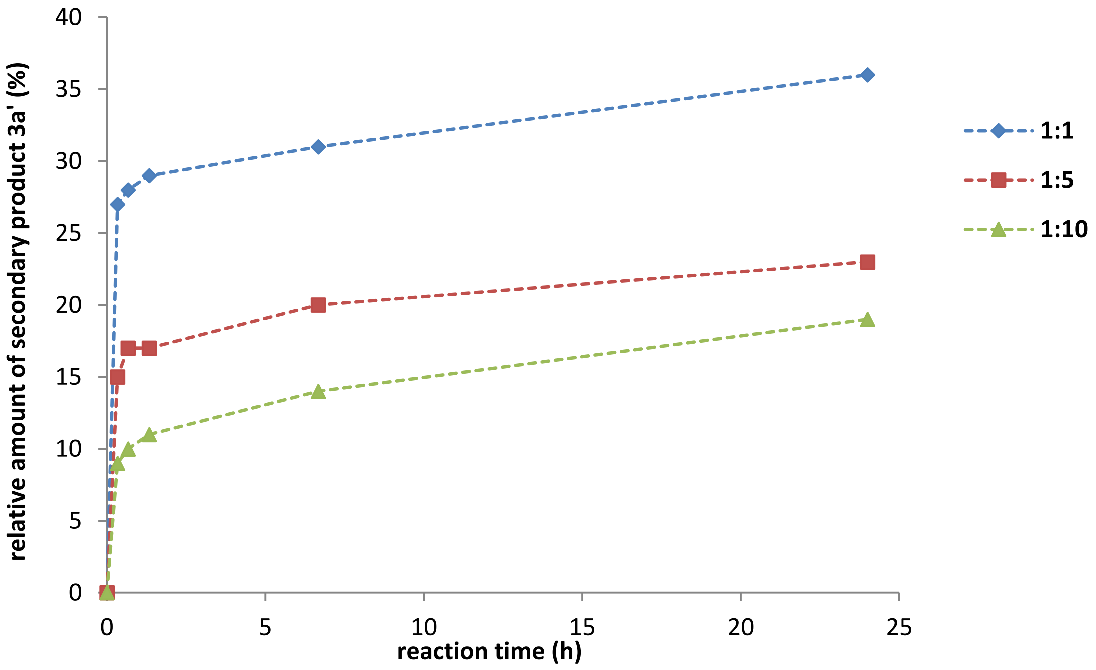


**Figure S1:** Percentage of secondary product for different time points using different substrate ratios. The carbonyl to hydrazine ratio was altered from 1:1 (blue) over 1:5 (red) to 1:10 (green). For this study the final concentration for *n*-butanal (**3**) was always set to 5 mM and the concentration for hydrazine (**a**) was increased from 5 mM to 50 mM.

## (B) Postulated reaction pathways:

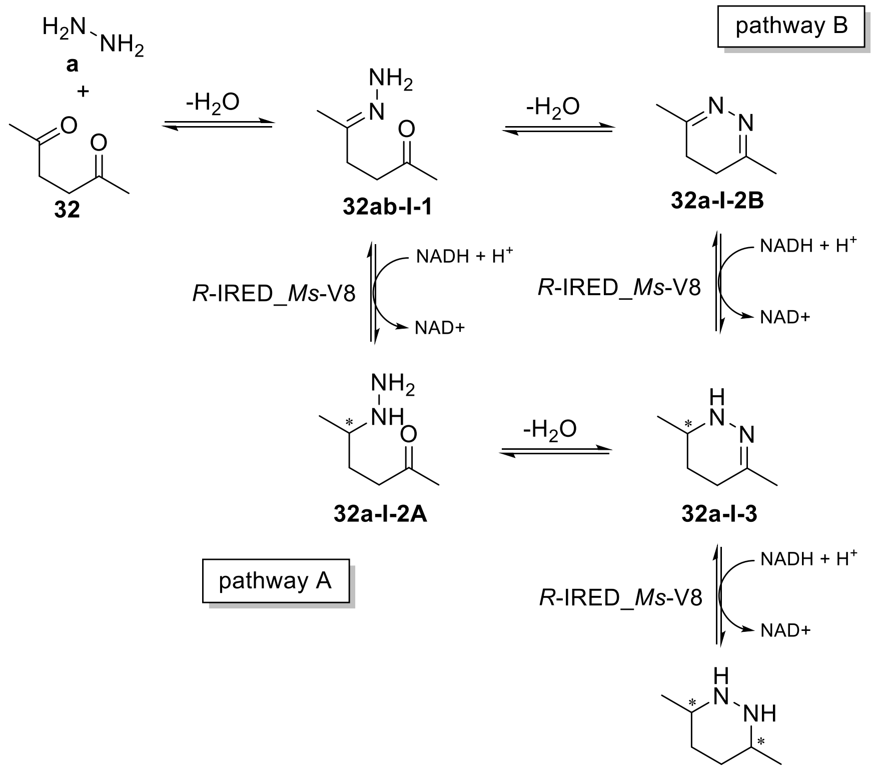


**Scheme S2:** Postulated reaction pathways for the double reductive hydrazination exemplarily for the formation of 32a. In pathway A, after the first (intermolecular) condensation the hydrazone intermediate (32a-I-1) is directly reduced by IRED. Then, a second (intramolecular) condensation forms the cyclic hydrazone intermediate (32a-I-3), which is reduced by IRED in the last step. In pathway B, both condensations take place before IRED reduces both C=N bonds. Results from previous studies regarding the double reductive amination indicate that *R*-IRED_*Ms*-V8 prefers pathway A.[1] This could also apply to the double reductive hydrazination.

## (C) Single reductive hydrazinations with TOF < $1^{min-1}$

**Table S2:** List of different products still being produced by the reductive hydrazination process but with TOF < 1 $min^{-1}$

| entry | Hydrazine product | TOF ($min^{-1}$) | entry | Hydrazine product | TOF ($min^{-1}$) |
|---|---|---|---|---|---|
| 1 | **5b** | 0.54 ± 0.05 | 8 | **19b** | 0.08 ± 0.02 |
| 2 | **6b** | 0.64 ± 0.10 | 9 | **20b** | 0.27 ± 0.02 |
| 3 | **7b** | 0.74 ± 0.02 | 10 | **21b** | 0.18 ± 0.01 |
| 4 | **8b** | 0.72 ± 0.04 | 11 | **24a** | 0.20 ± 0.04 |
| 5 | **13b** | 0.31 ± 0.03 | 12 | **24b** | 0.83 ± 0.17 |
| 6 | **15b** | 0.24 ± 0.02 | 13 | **24c** | 0.19 ± 0.01 |
| 7 | **16b** | 0.55 ± 0.03 | 14 | **25b** | 0.51 ± 0.15 |

# 3 General procedures

**(A) R-IRED_Ms-V8 protein expression, and purification:**

*E. coli* JW5510 cells were transformed with a pBAD33 plasmid harboring the gene for *R*-IRED_*Ms*-V8 with an *N*-terminal $his_6$-tag. The transformed cells were grown over night at 37°C. Terrific broth (TB) culture medium containing 34 µg $mL^{-1}$ chloramphenicol was inoculated using 0.25% *v/v* of preculture. After 2-3 h of incubation at 37°C and reaching an optical density of $OD_{600} \approx 1.0$, the protein expression was induced with the addition of arabinose (final concentration 0.02%). The cultures were incubated for 20 h at 25°C. After harvesting and lysing the cells, protein purification was performed with Co loaded His-Trap columns (His-GraviTrap-TALON, GE Healthcare) using 50 mM potassium phosphate buffer pH 7.0, 300 mM KCl for binding, 50 mM potassium phosphate buffer pH 7.0, 300 mM KCl, 5 mM imidazole for washing and 50 mM potassium phosphate buffer pH 7.0, 300 mM KCl, 500 mM imidazole for eluting the enzyme. After purification, the buffer was changed via dialysis (two times for 2 h in 5 L, 50 mM potassium phosphate buffer pH 7.0, 6-8 MWCO). Purity and size were verified by SDS-PAGE. The protein concentration was determined using the BCA Protein Assay Kit (Thermo Scientific).

**(B) Soluble hydrogenase (SH) protein production and purification:**

*Cupriavidus necator* cells harboring the pGE771 plasmid[2] were cultivated heterotrophically in mineral salts medium supplemented with 0.05% (w/v) fructose, 0.4% (v/v) glycerol, 1 µM $NiCl_2$, 40 µM $FeCl_3$, 1 µM $ZnCl_2$, and trace element solution. Cultures were grown in 5 L Erlenmeyer flasks, shaking at 120 rpm until reaching an $OD_{436}$ of approximately 11. Cells were then harvested by centrifugation at 6000 g for 12 minutes at 4°C. After harvesting, the cells were resuspended in 50 mM potassium phosphate buffer pH 7.0, containing 5% glycerol, 5 mM $NAD^+$, and an EDTA-free protease inhibitor cocktail (Roche), in a 3:1 $buffer_{ml}$:$cells_g$ ratio. The suspension was degassed with argon before cell disruption by cooled French press at 18 kPSI. The lysate was centrifuged at 100,000g for 45 minutes, with the supernatant kept under an argon atmosphere to preserve the soluble proteins. This extract was applied to a Strep-Tactin Superflow column (IBA) and washed with 12 mL of the resuspension buffer, followed by 18 mL of the $NAD^+$-free buffer. The SH protein was then eluted using the $NAD^+$-free buffer containing 5 mM desthiobiotin. Eluted fractions with SH protein were pooled and concentrated using an Amicon Ultra-15 device (100 MWCO, Merck Millipore, Germany). Purity and size was verified by SDS-PAGE. The protein concentration was determined using the BCA Protein Assay Kit (Thermo Scientific).

**(C) NADH depletion assay for determining enzyme activity**

Enzyme activity was quantified using a spectrophotometric assay. The enzyme and the substrates were put into a microtiter plate to a volume of 180 µl. The reaction was started directly before the measurement by the addition of 20 µl NADH (4 mM) to get a final volume of

200 µl and a final NADH concentration of 0.4 mM. NADH consumption was monitored at a wavelength (λ) of 340 nm, with readings taken every 20 seconds over a period of 20 minutes.

**(D) Biotransformations for the reductive hydrazination:**

Biotransformations were performed on a 250 µL scale in glass vials. The reaction mixture included 15 µM of purified IRED, 5 mM of the carbonyl substrate, 5 mM hydrazine, 2 mM NADH, 2.5 mM $MgCl_2$, 30 mM glucose-6-phosphate, 5 U $mL^{-1}$ glucose-6-phosphate dehydrogenase. The buffer system used was 100 mM potassium phosphate buffer at pH 6.0. The reaction mixture was incubated at 25°C for 4 hours, after which the reaction was terminated by the addition of 20 µL of 5 M aqueous NaOH.

**(E) Biotransformations for the double reductive hydrazination:**

Biotransformations were performed on a 250 µL scale in glass vials. The reaction mixture included 15 µM of purified IRED, 5 mM of the carbonyl substrate, 5 mM hydrazine, 2 mM NADH, 2.5 mM $MgCl_2$, 30 mM glucose-6-phosphate, 5 U $mL^{-1}$ glucose-6-phosphate dehydrogenase. The buffer system used was 100 mM potassium phosphate buffer at pH 6.0. The reaction mixture was incubated at 25°C for 4 hours, after which the reaction was terminated by the addition of 20 µL of 5 M aqueous NaOH.

**(F) Biotransformations for the $H_2$-driven double reductive hydrazination:**
Biotransformations were conducted in 10 mL scale in non-gas permeable glass septum tubes. The reaction mixture comprised 15 µM of purified IRED, 5 mM of the carbonyl substrate, 5 mM hydrazine, 0.4 mM NAD+, and 2.5 µM of purified SH. A buffer system of 100 mM potassium phosphate at pH 7.0 was utilized. The mixture was incubated at 25°C for 8 hours, and the reaction was halted by adding 20 µL of 5 M aqueous NaOH.

To maintain an optimal reaction environment, the system was saturated with $H_2$. All components were added using a Hamilton gas-tight syringe through the septum, ensuring the preservation of $H_2$ saturation conditions throughout the process.

**(G) Sample preparation and derivatization for GC-MS or GC-FID analysis:**

For GC analysis, 100 µL of each sample was quenched with 10 µL of 5 M NaOH(aq). Subsequently, the samples underwent simultaneous extraction and derivatization. This was achieved by adding 250 µL of derivatization mix (comprising 600 mM acetic anhydride in dichloromethane) and vortexing for 1 minute. The resulting organic phase was then subjected to gas chromatography for analysis.

In cases where derivatization was not required for certain products, the protocol was modified: 250 µL of dichloromethane was used in place of the derivatization mix.

# 4 GC chromatograms

## (A) GC-MS analysis for product identification:

**Table S3:** Retention times (min) of detected hydrazine products for the GC-MS. Most products were detected in their acetylated form.

| Product | Temperature program | Retention time (min) |
|---|---|---|
| **2b** | A | 4.75, 5.17 |
| **3a** | A | 6.70, 6.83, 7.49 |
| **3b** | B | 6.57 |
| **3c** | A | 4.33 |
| **3g*** | B | 5.25 |
| **4b** | A | 6.16 |
| **11b*** | A | 4.94, 5.14 |
| **12b** | A | 8.40 |
| **14b** | B | 5.59 |
| **24a*** | A | 5.24 |
| **24b*** | B | 6.29 |
| **24c*** | A | 4.85 |
| **28a** | A | 6.85 |
| **28b** | B | 6.20, 6.88 |
| **28c** | A | 5.85 |
| **28d*** | A | 6.14, 6.29 |
| **28e*** | A | 6.91 |
| **28g*** | B | 6.38 |
| **32a** | A | 4.90, 6.05 |
| **32b** | A | 4.88, 4.96 |
| **32c*** | C | 5.85 |
| **33a** | A | 7.23 |
| **34a** | A | 4.67, 6.04 |
| **34b** | B | 6.24, 6.27 |
| **34c*** | C | 5.00 |
| **34f*** | A | 6.86 |
| **34g** | B | 5.94 |

* Derivatization was omitted.

**(B) GC-MS Chromatograms and mass spectra for hydrazine products listed in table S3.**

GC-MS data – biotransformation **2b**

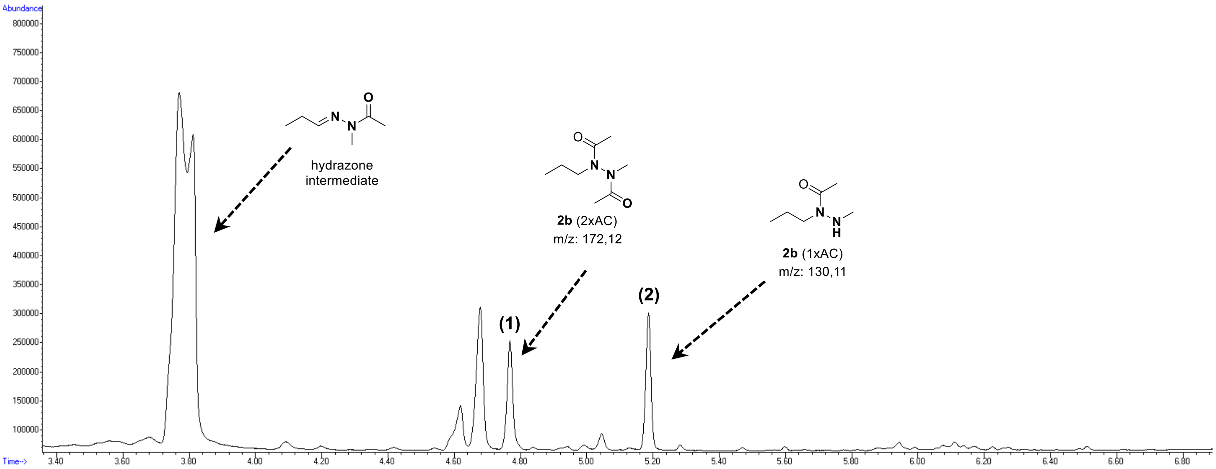


Mass spectrum of **2b** (1):

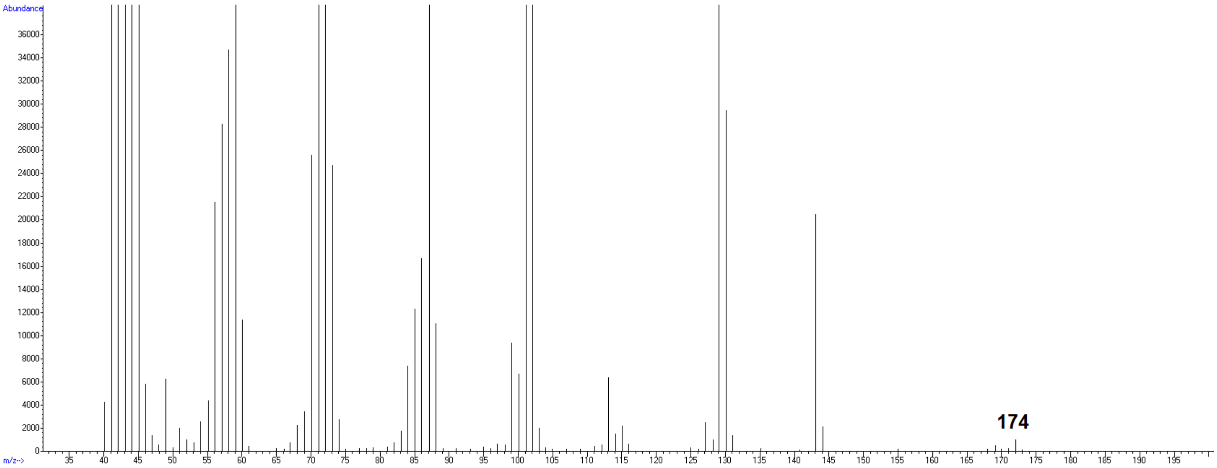


Mass spectrum of **2b** (2):

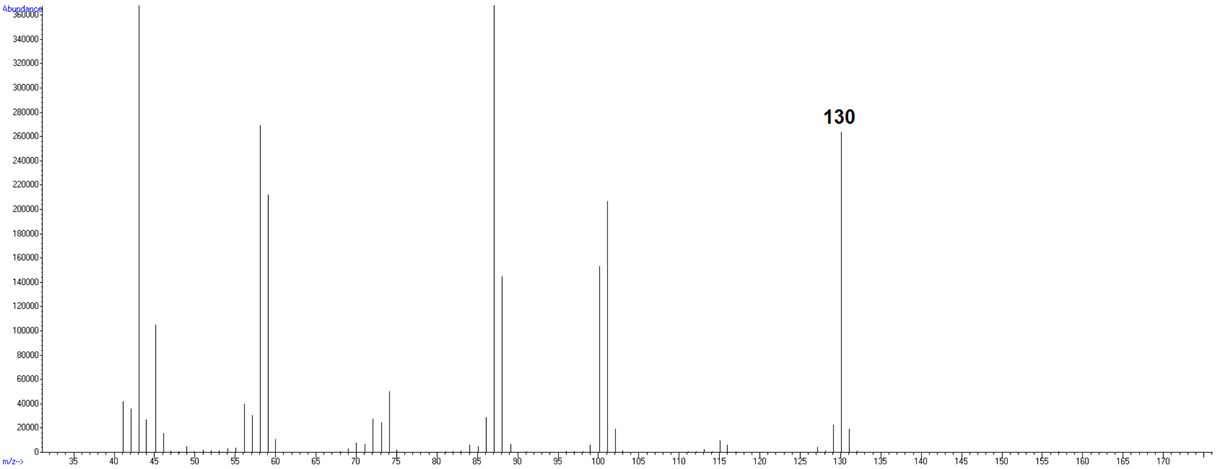

<u>GC-MS data – biotransformation **3a**</u>

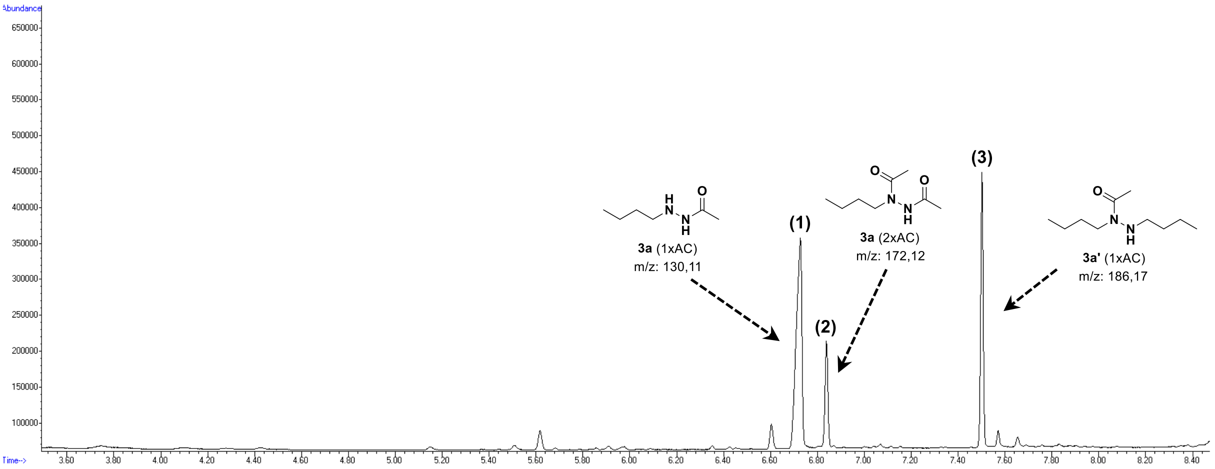


Mass spectrum of **3a** (1):

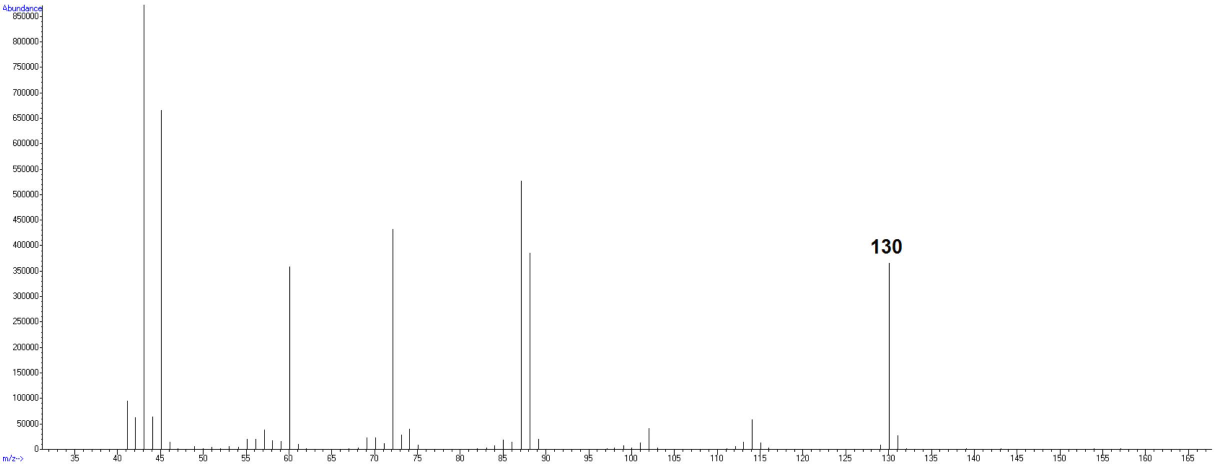


Mass spectrum of **3a** (2):

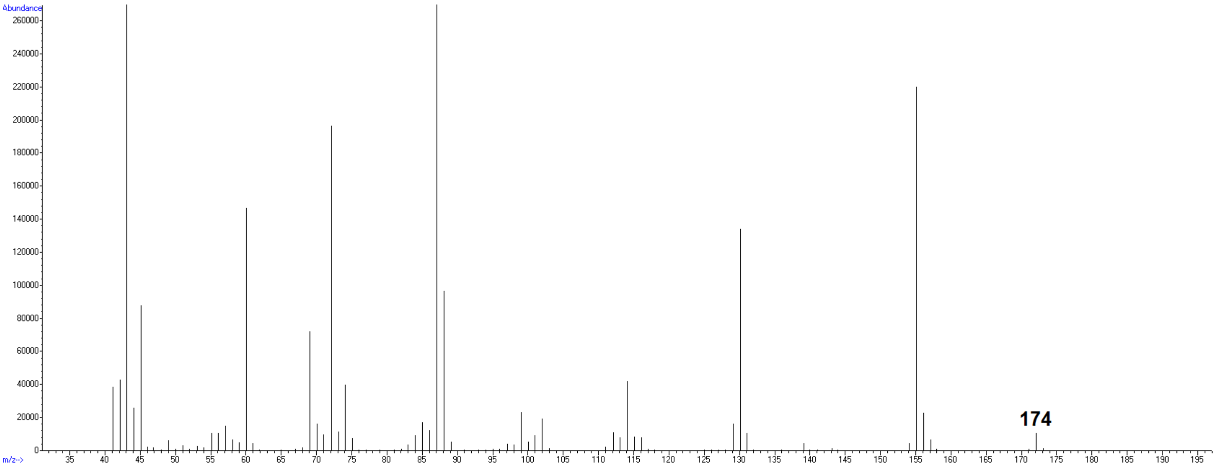

Mass spectrum of **3a'** (3):

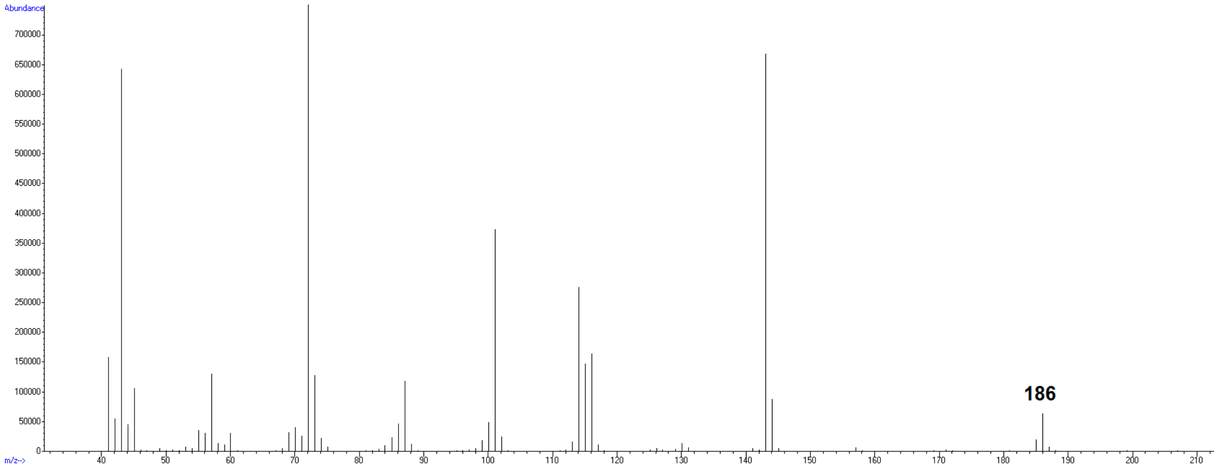


<u>GC-MS data – biotransformation **3b**</u>

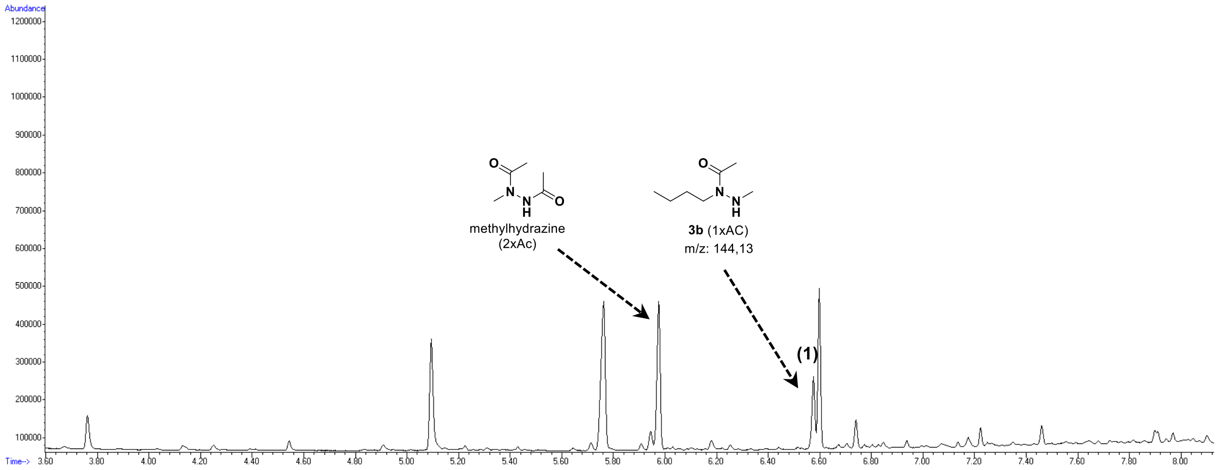


Mass spectrum of **3b** (1):

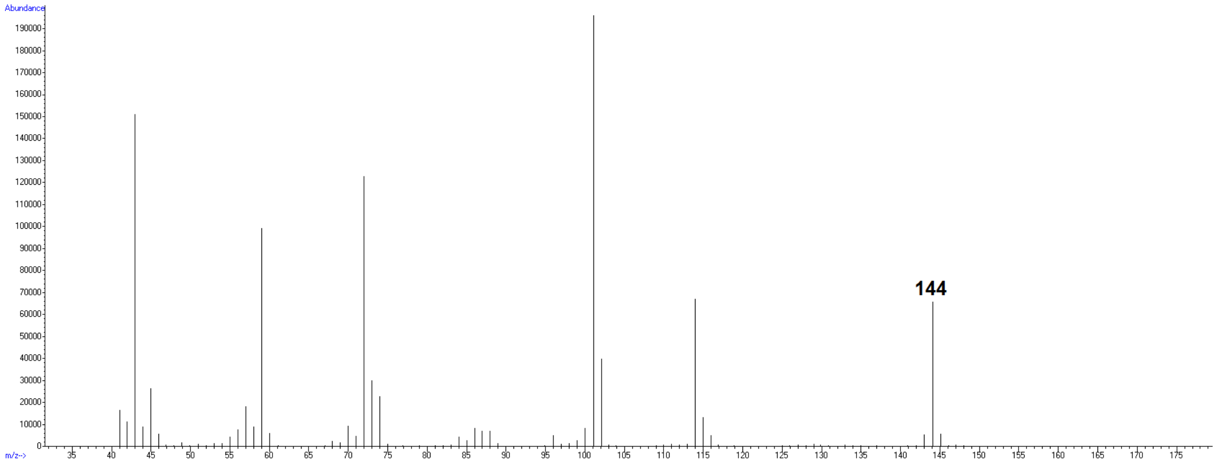

GC-MS data – biotransformation **3c**

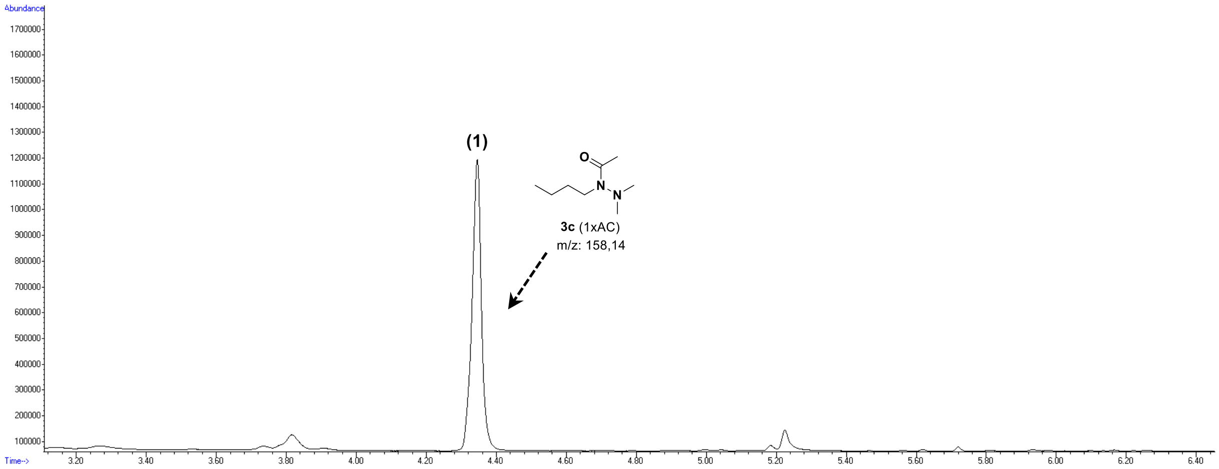


Mass spectrum of **3c** (1):

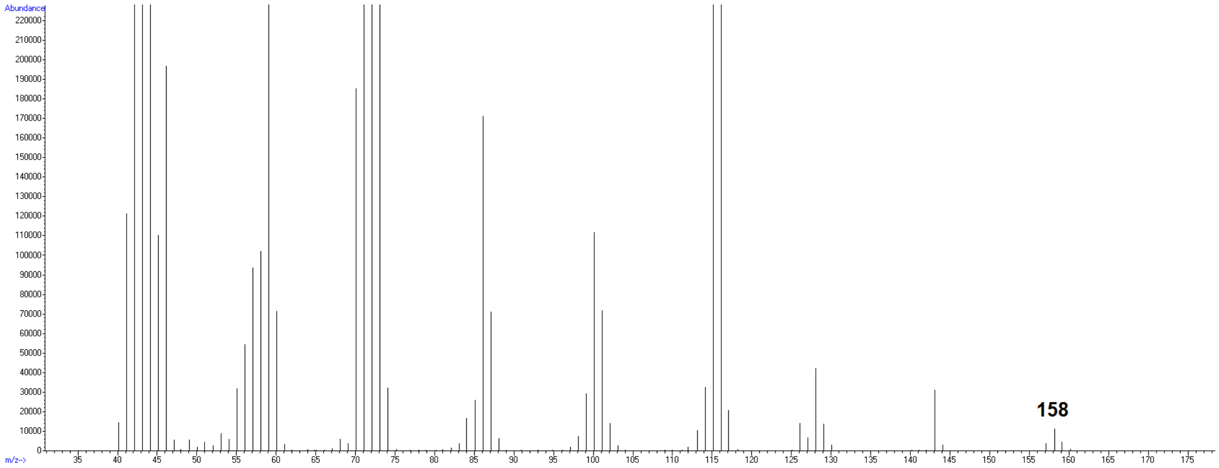


GC-MS data – biotransformation **3g**

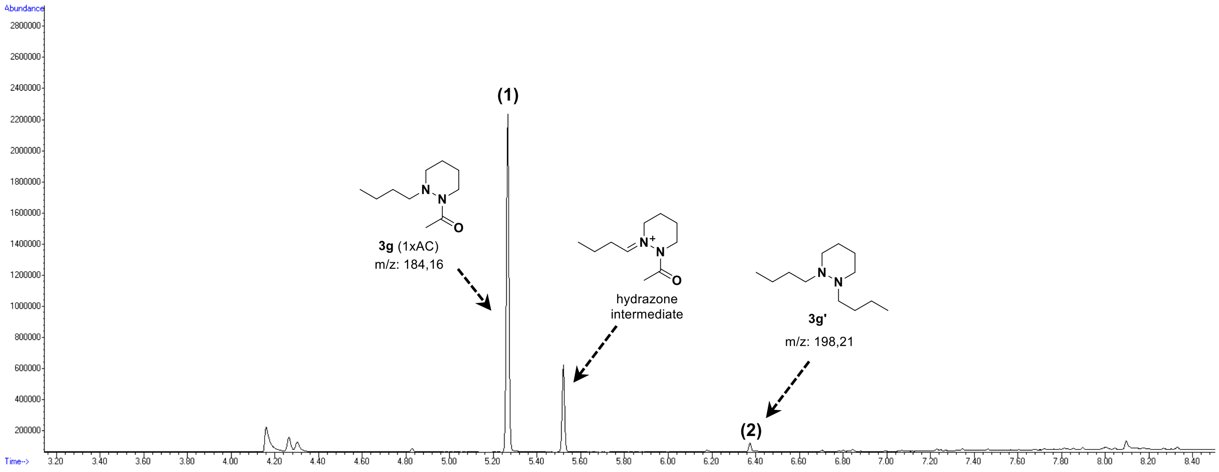

Mass spectrum of **3g** (1):

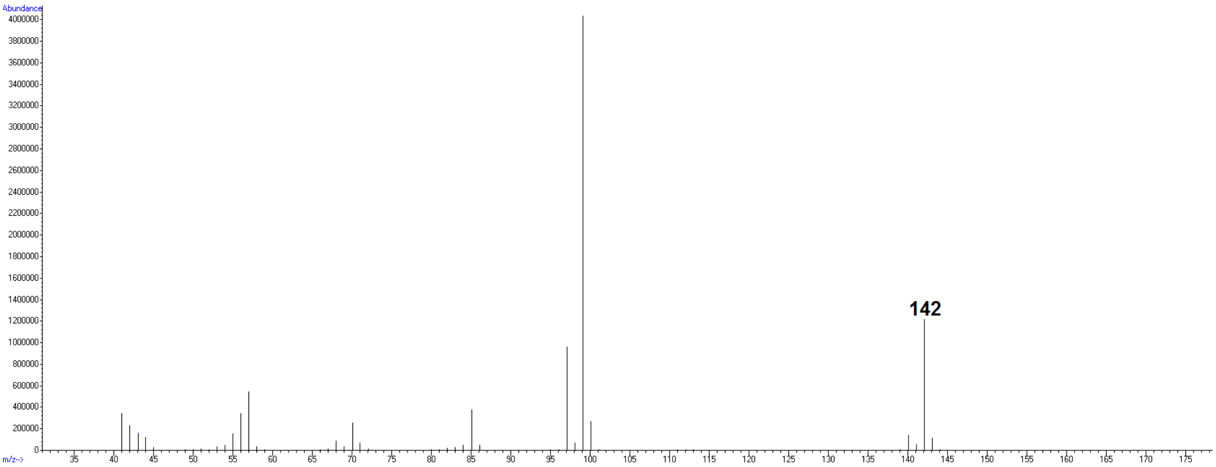


Mass spectrum of **3g'** (2):

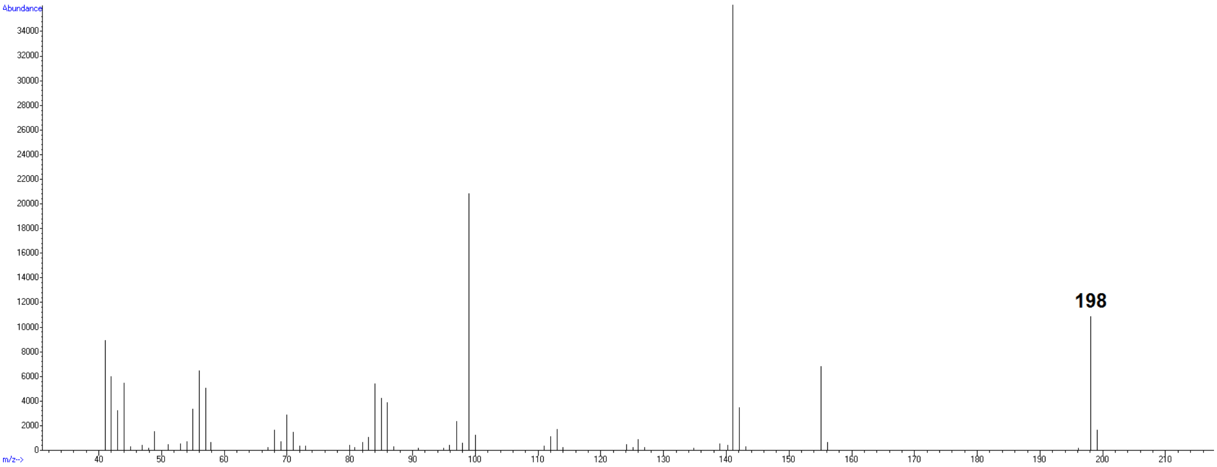


GC-MS data – biotransformation **4b**

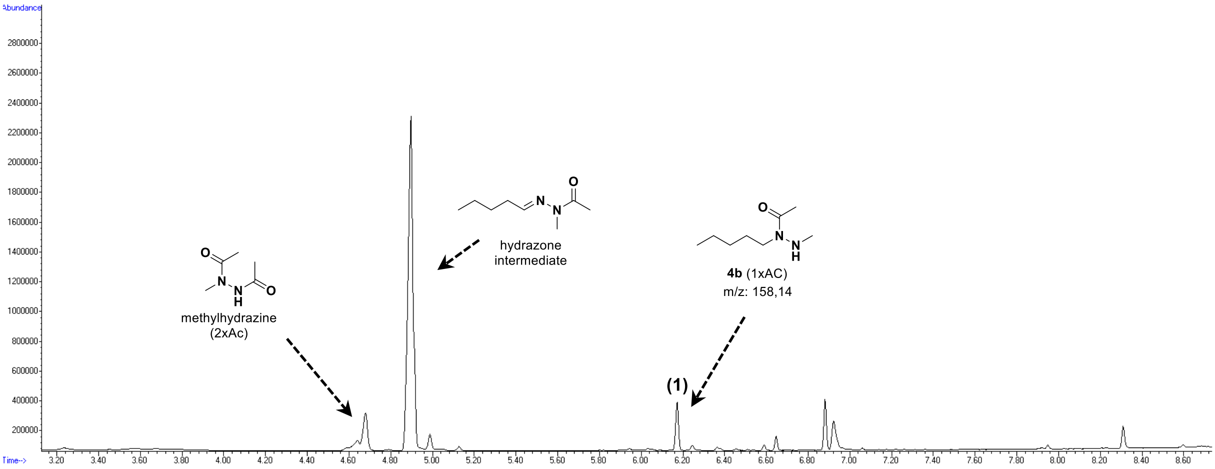

Mass spectrum of **4b** (1):

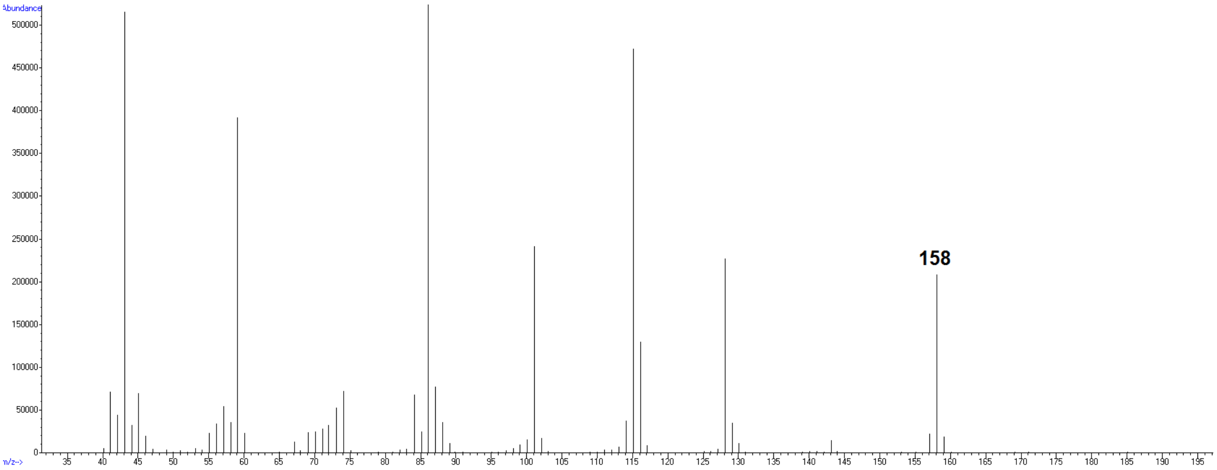


<u>GC-MS data – biotransformation **11b**</u>

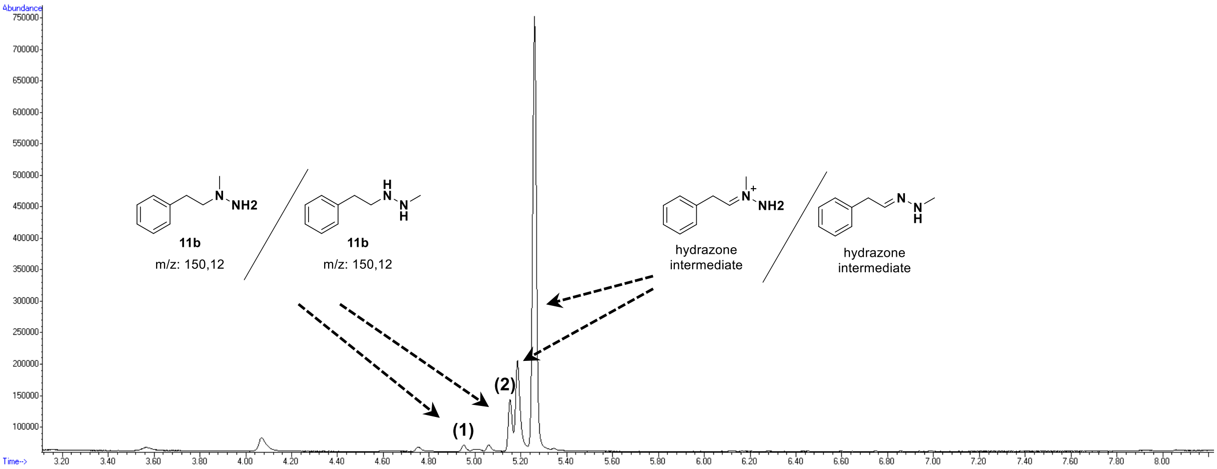


Mass spectrum of **11b** (1):

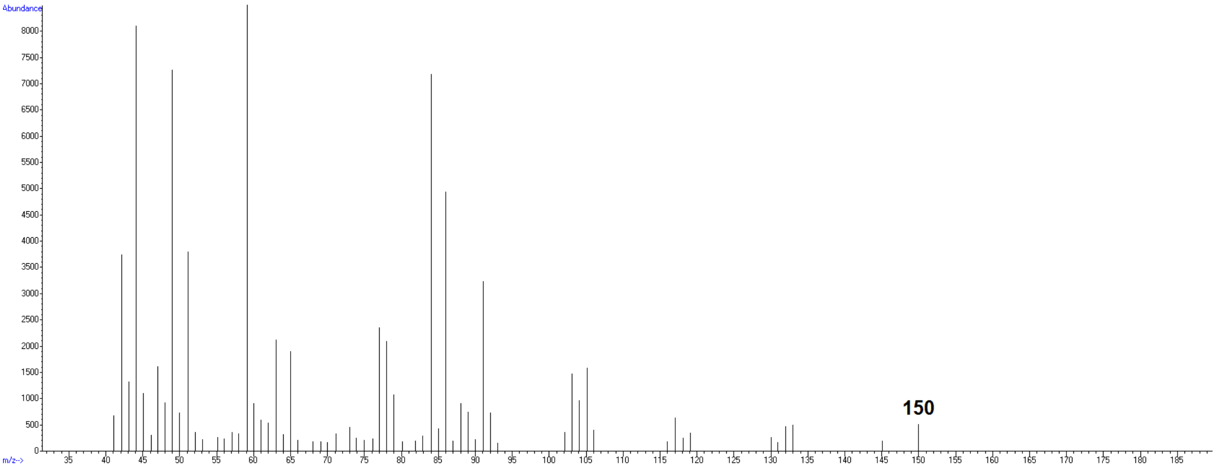

Mass spectrum of **11b** (2):

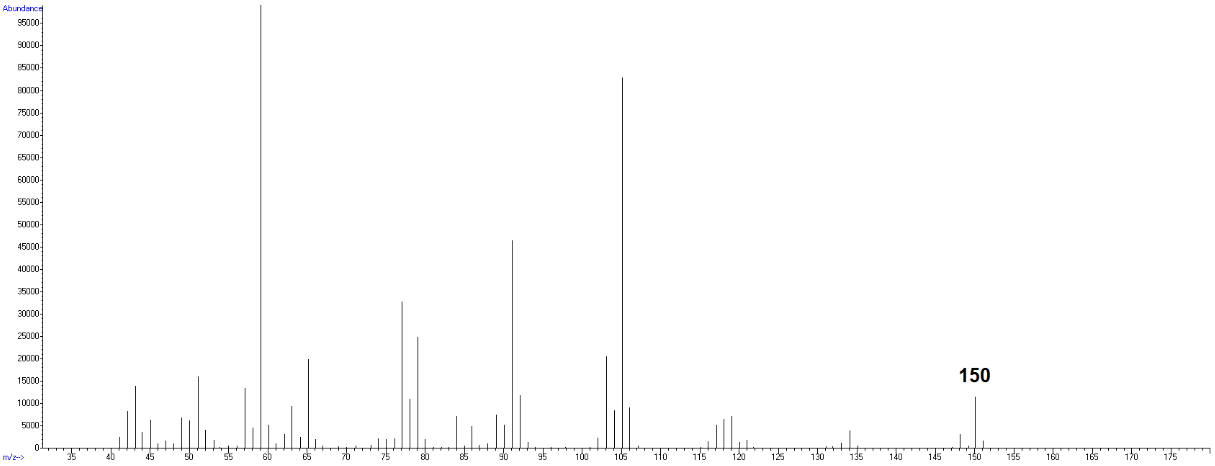


<u>GC-MS data – biotransformation **12b**</u>

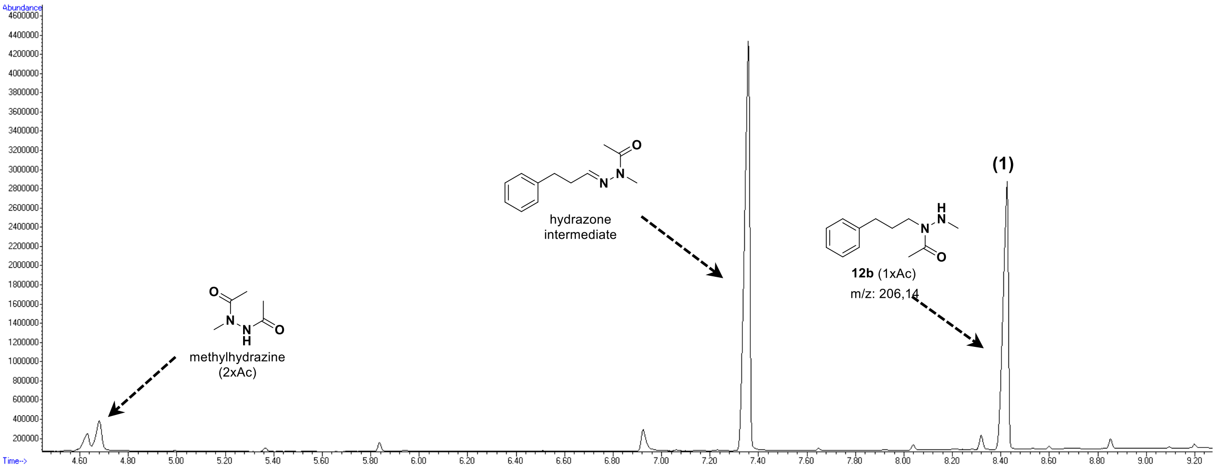


Mass spectrum of **12b** (1):

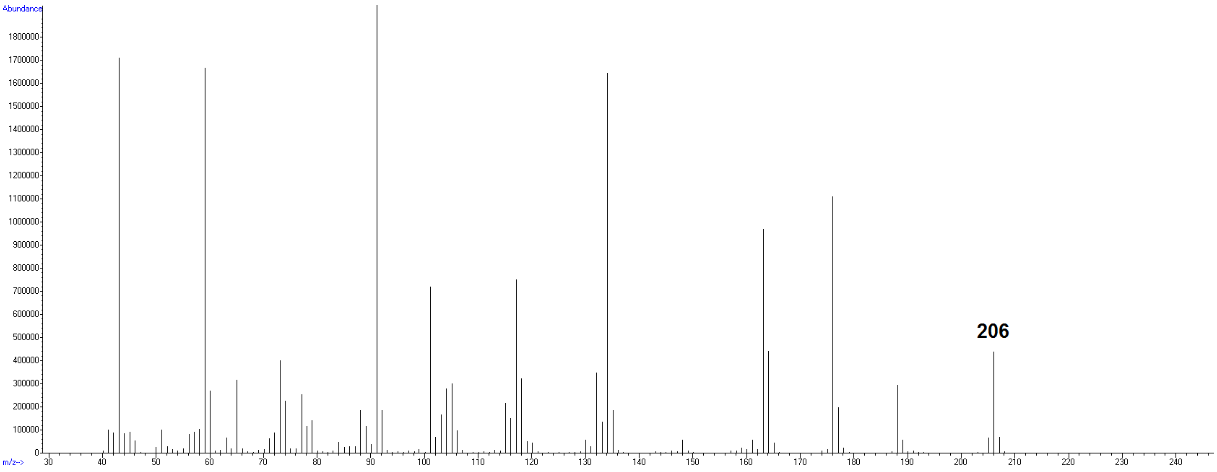

GC-MS data – biotransformation **14b**

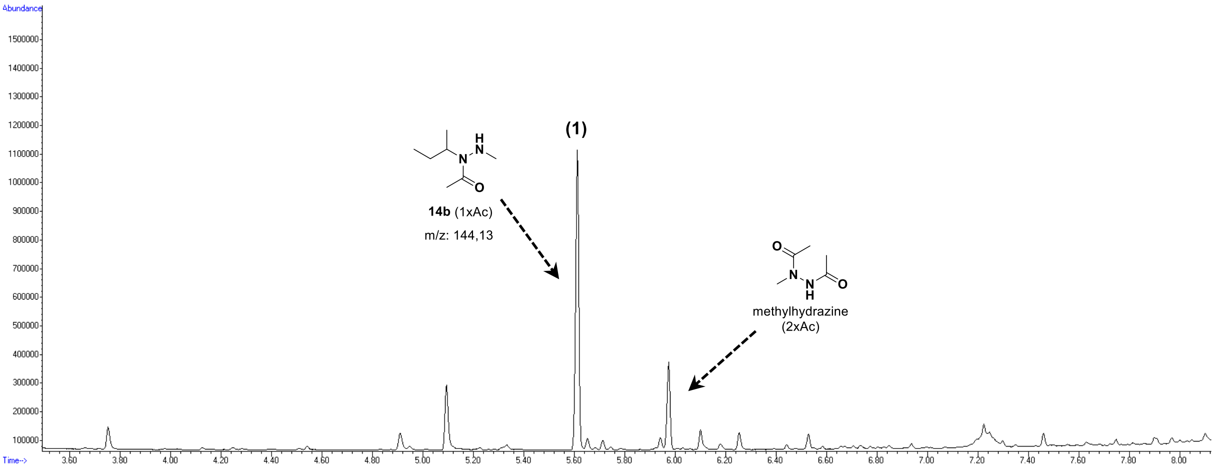


Mass spectrum of **14b** (1):

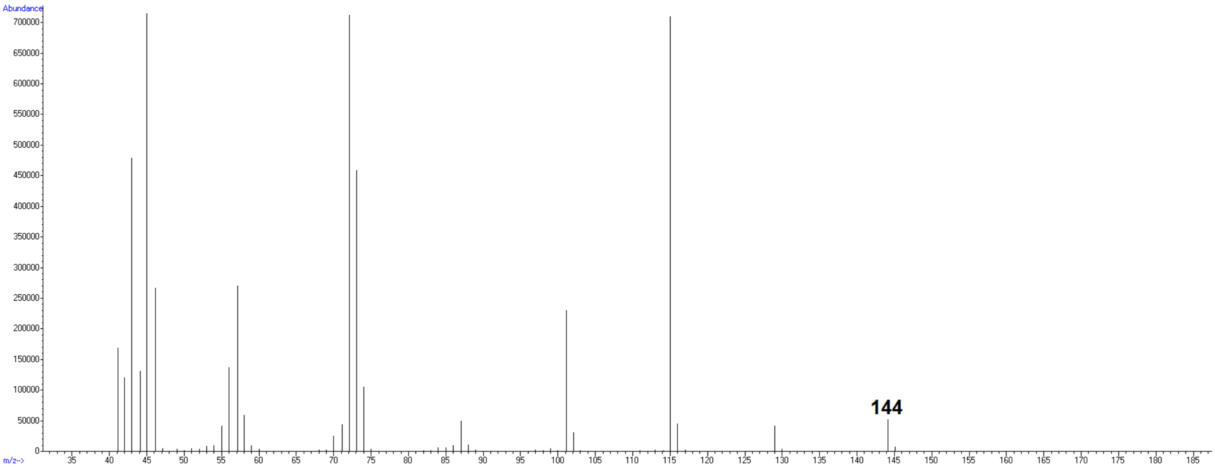


GC-MS data – biotransformation **24a**

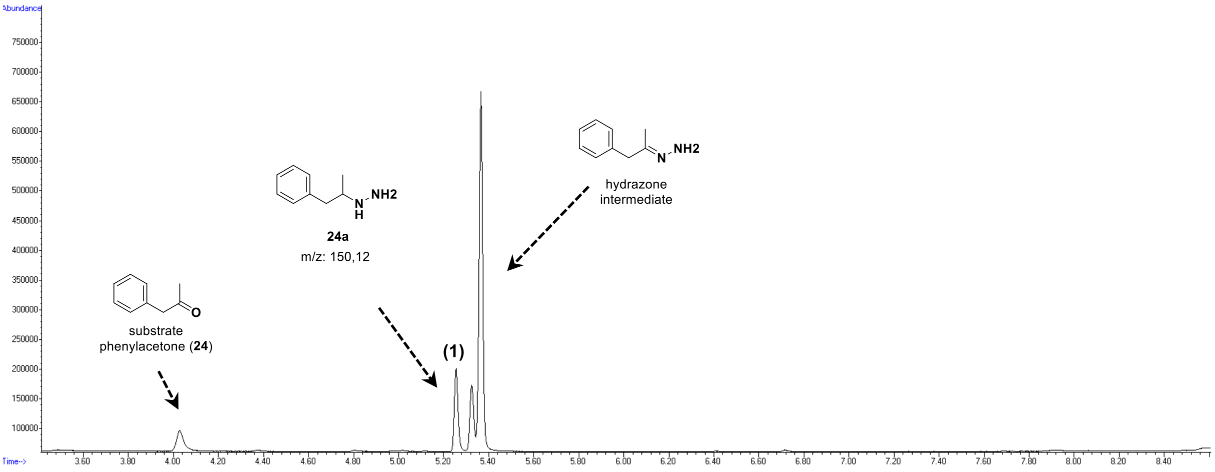

Mass spectrum of **24a** (1):

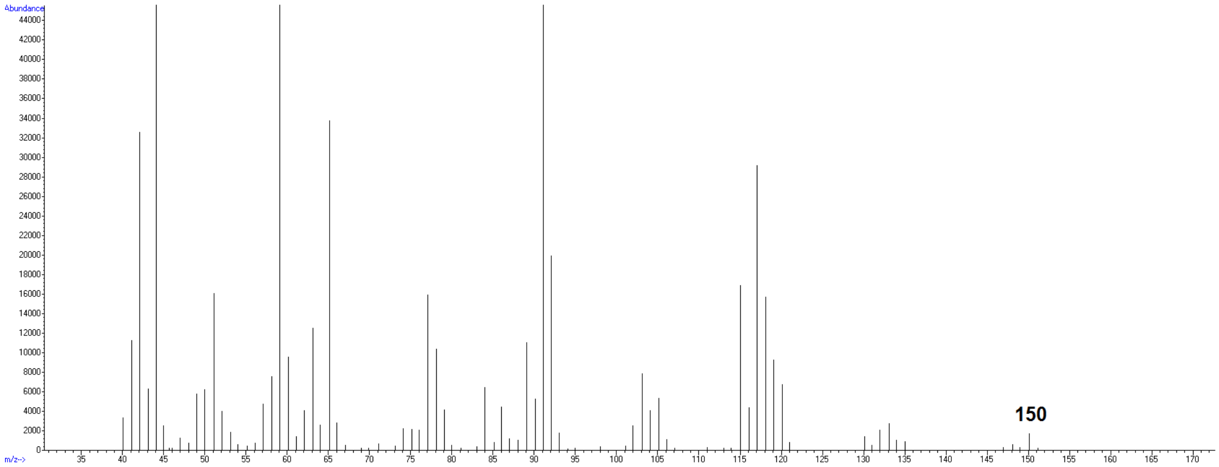


<u>GC-MS data – biotransformation **24b**</u>

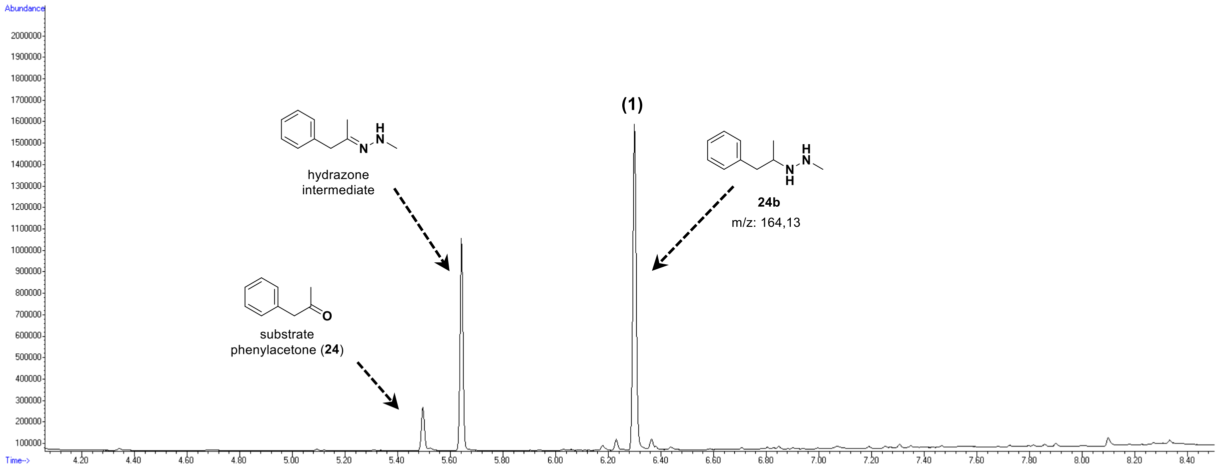


Mass spectrum of **24b** (1):

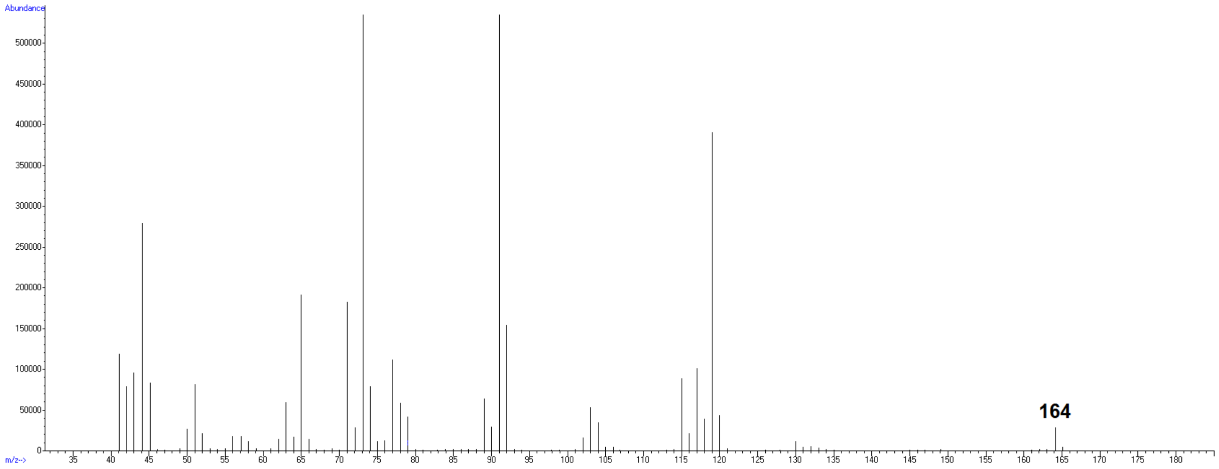

## GC-MS data – biotransformation **24c**

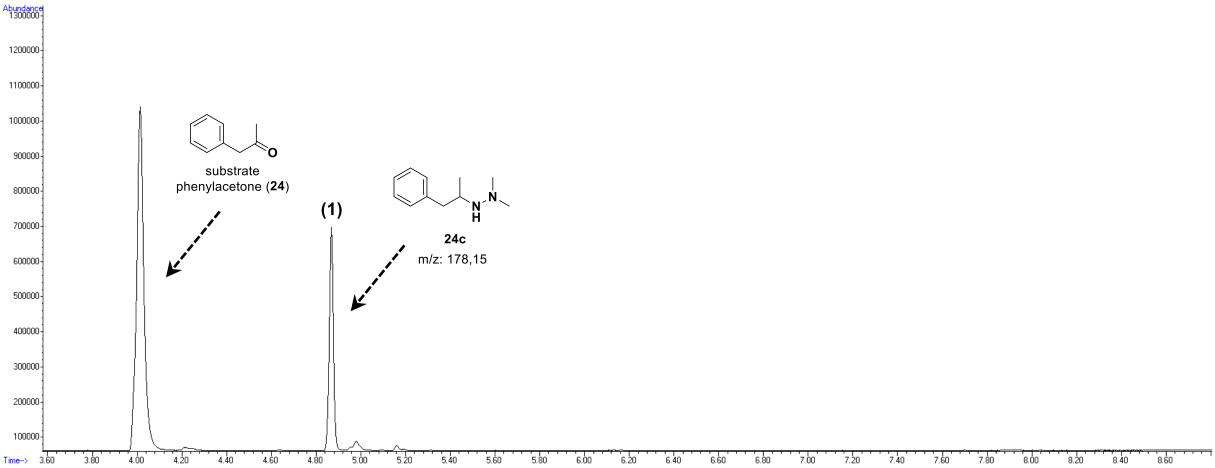


## Mass spectrum of **24c** (1):

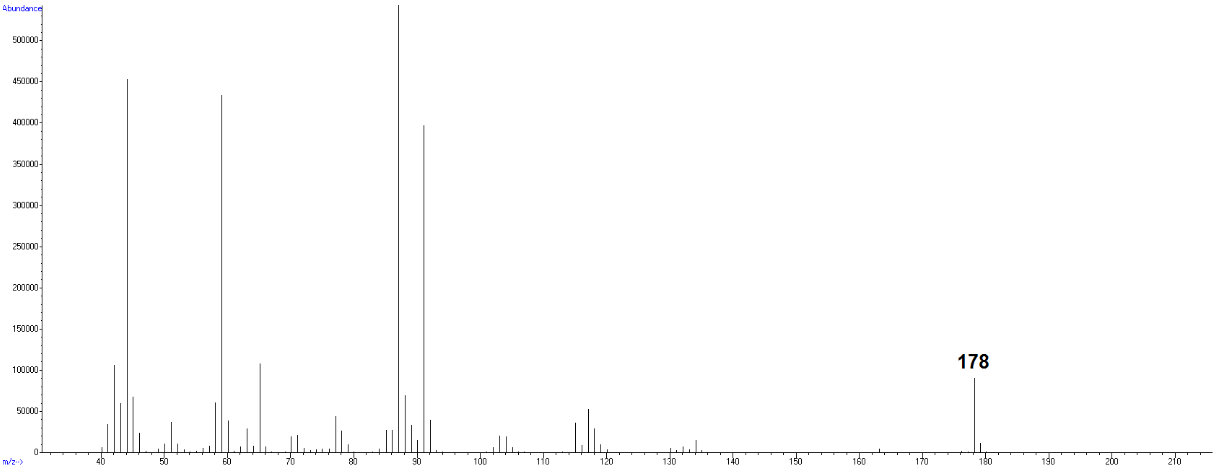


## GC-MS data – biotransformation **28a**

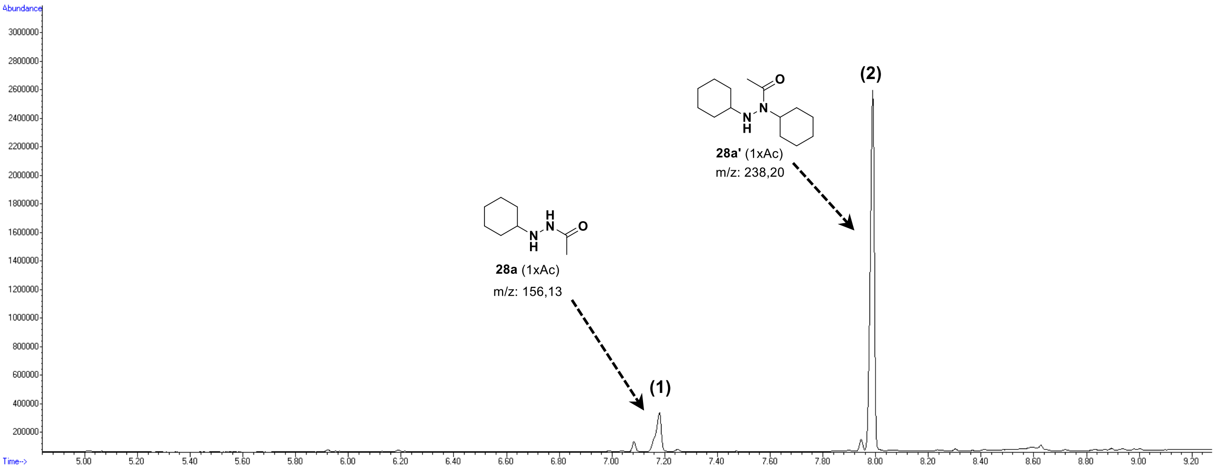

Mass spectrum of **28a** (1):

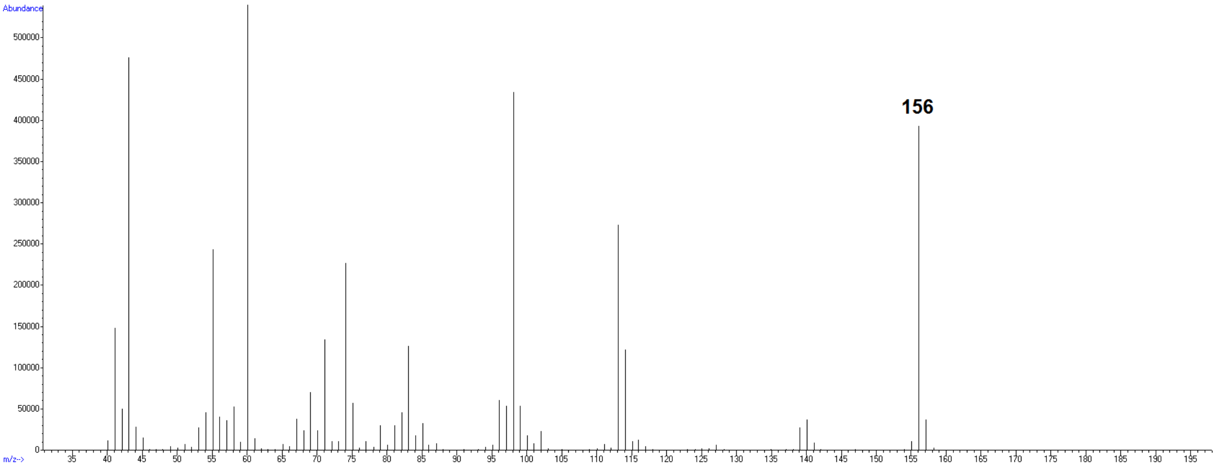


Mass spectrum of **28a'** (2):

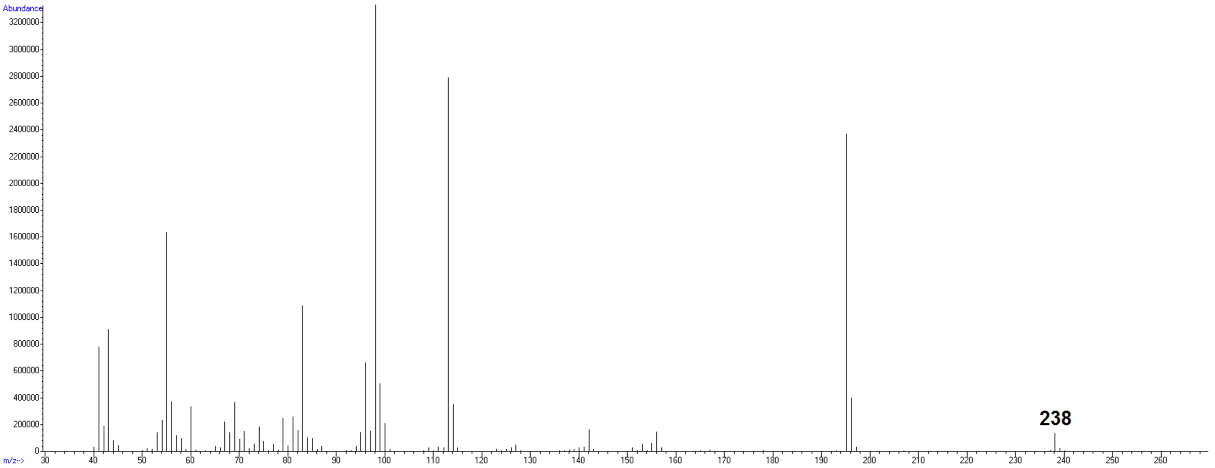


## <u>GC-MS data – biotransformation **28b**</u>

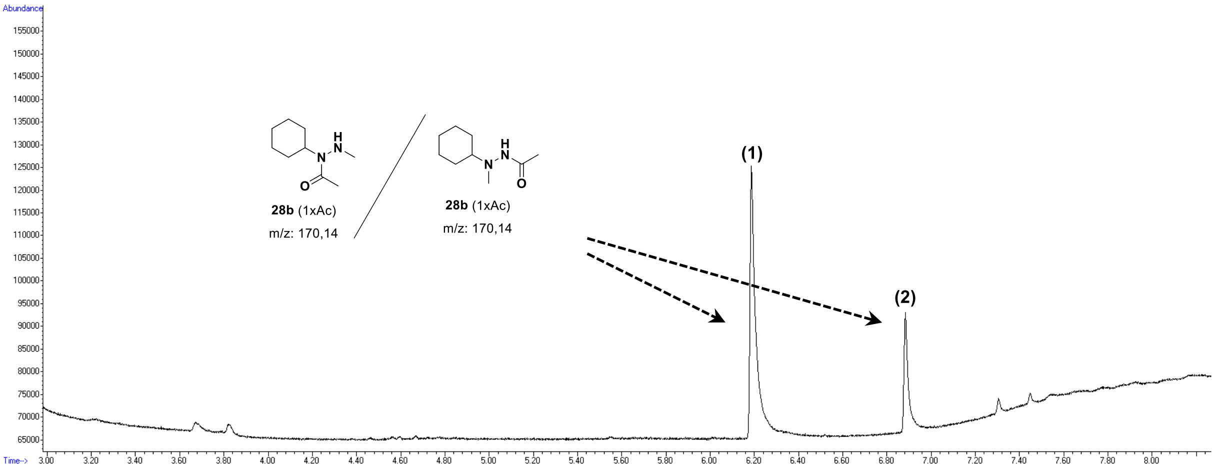

Mass spectrum of **28b** (1):

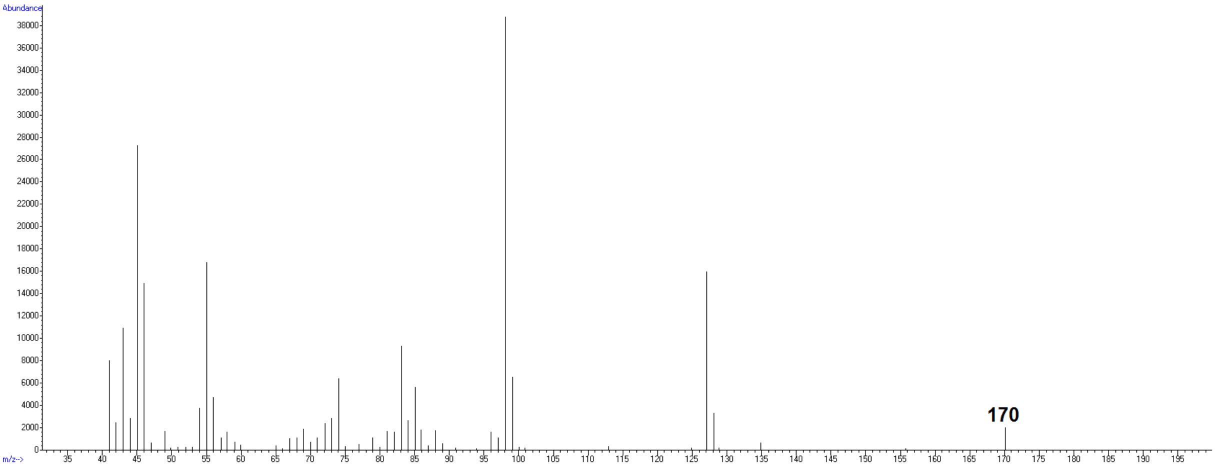


Mass spectrum of **28b** (2):

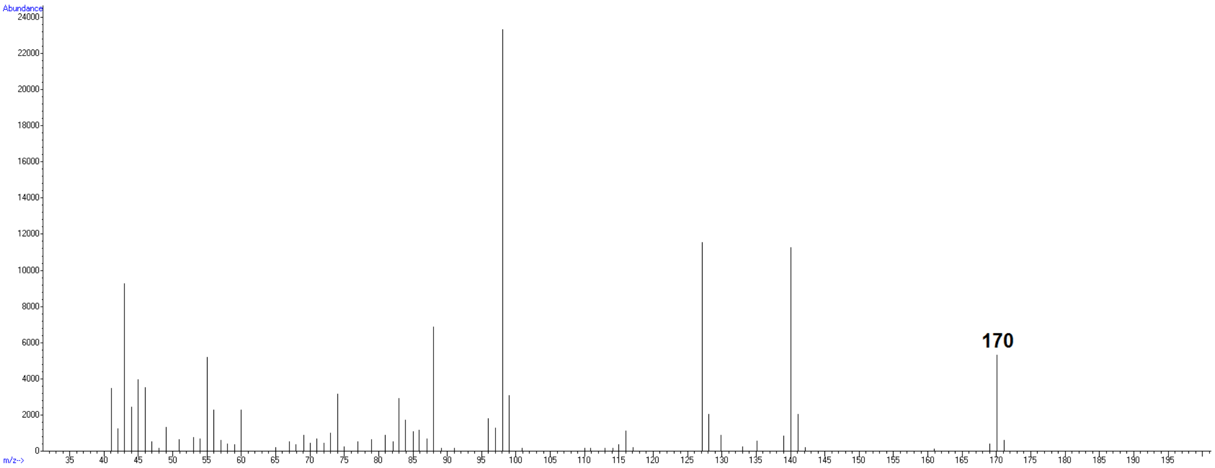


<u>GC-MS data – biotransformation **28c**</u>

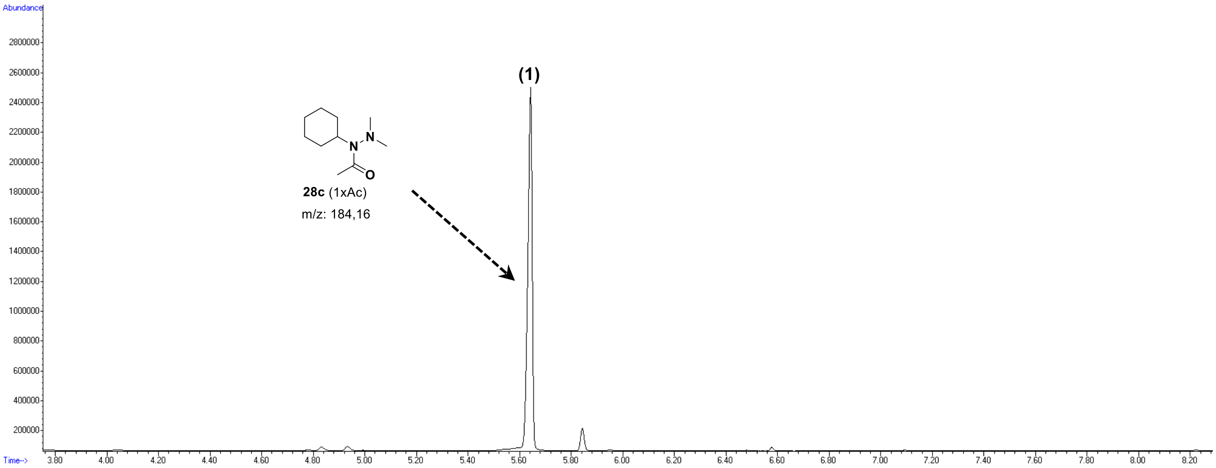

Mass spectrum of **28c** (1):

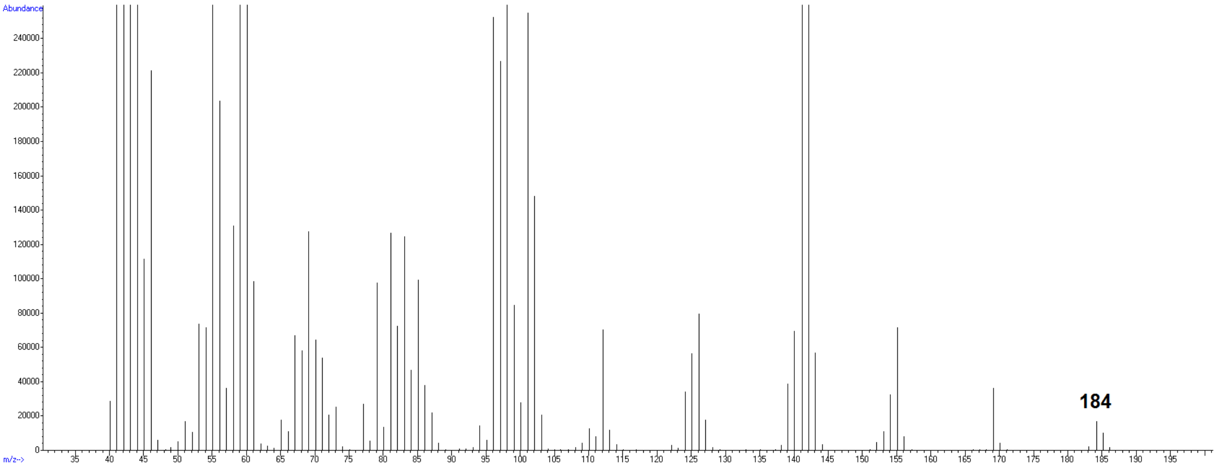


## <u>GC-MS data – biotransformation **28d**</u>

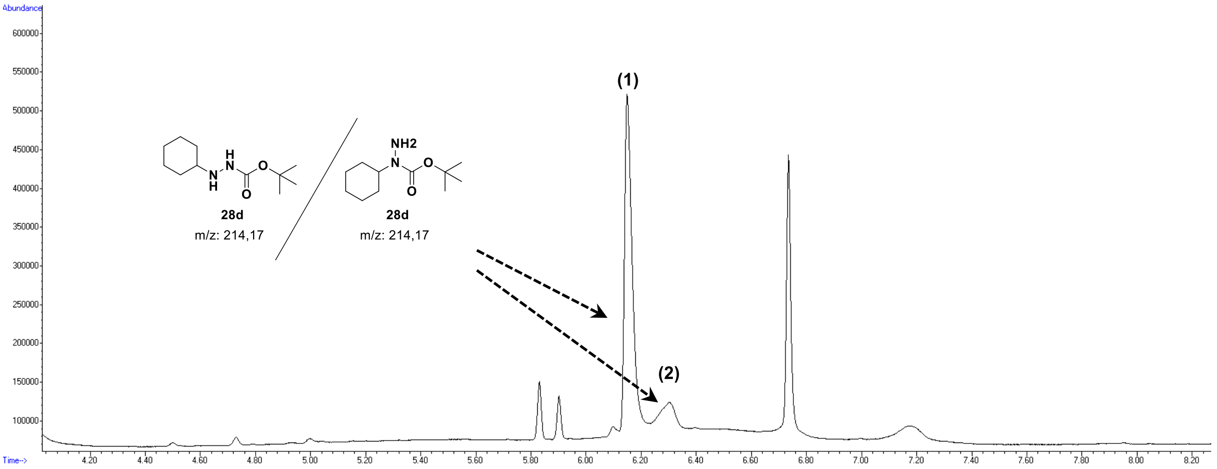


Mass spectrum of **28d** (1):

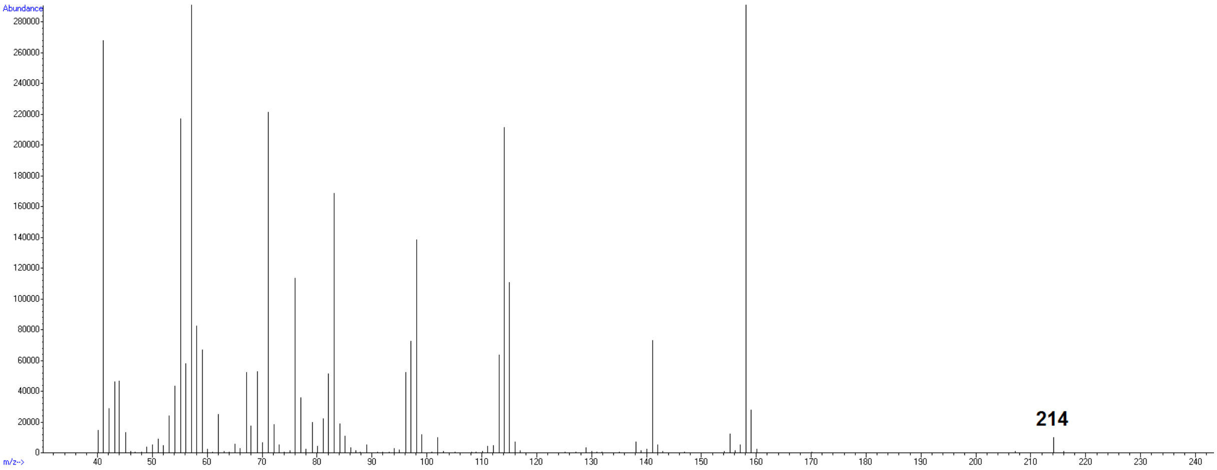

Mass spectrum of **28d** (2):

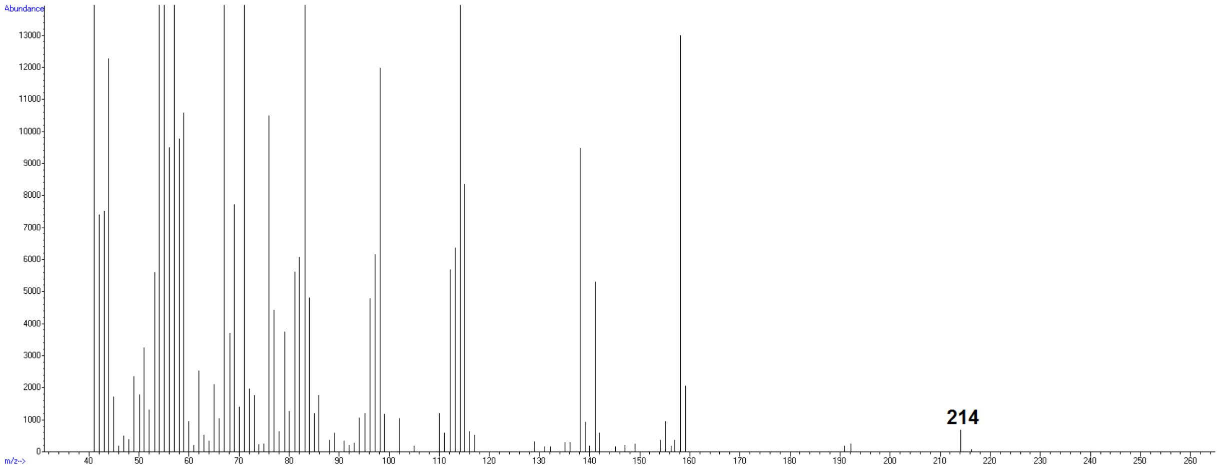


<u>GC-MS data – biotransformation **28e**</u>

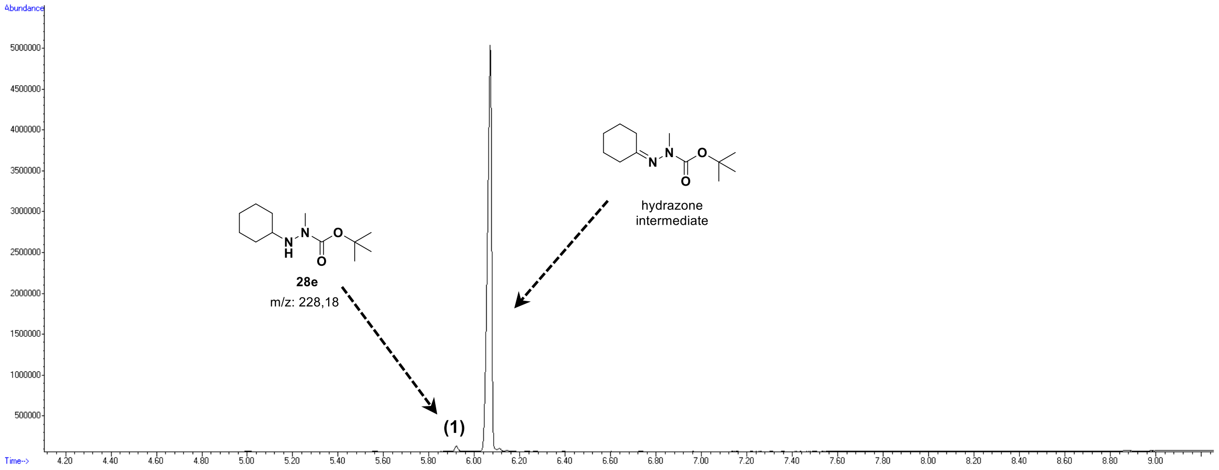


Mass spectrum of **28e** (1):

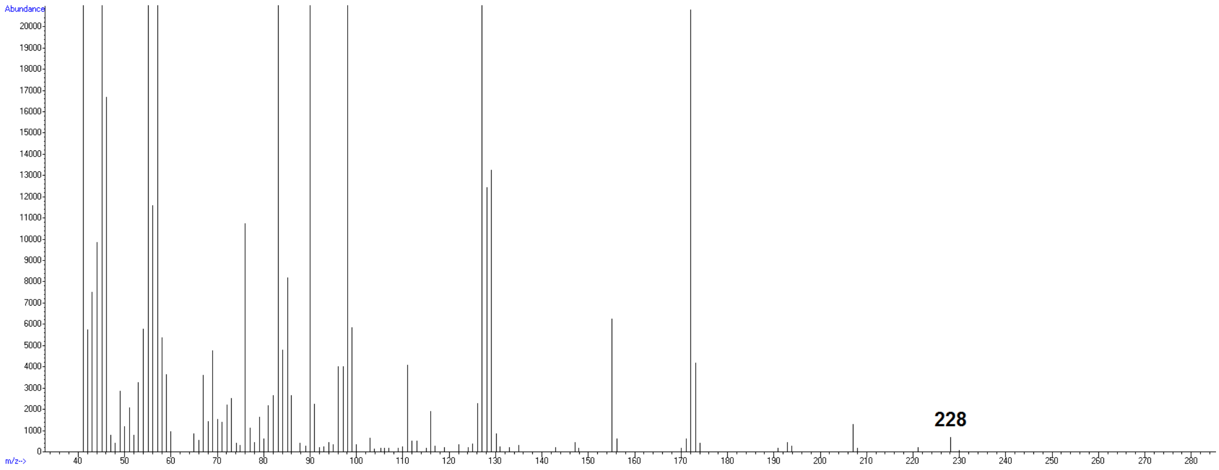

## GC-MS data – biotransformation **28g**

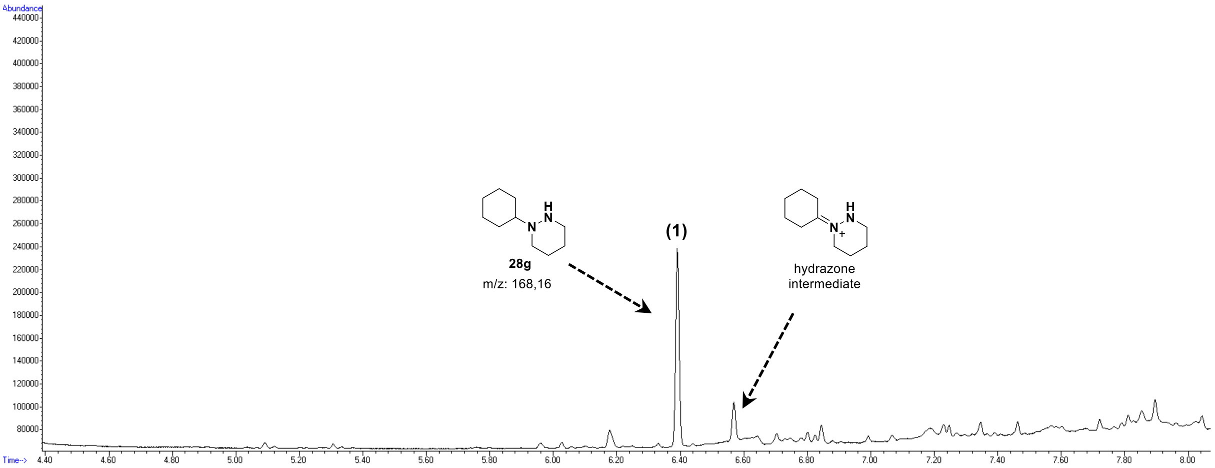


## Mass spectrum of **28g** (1):

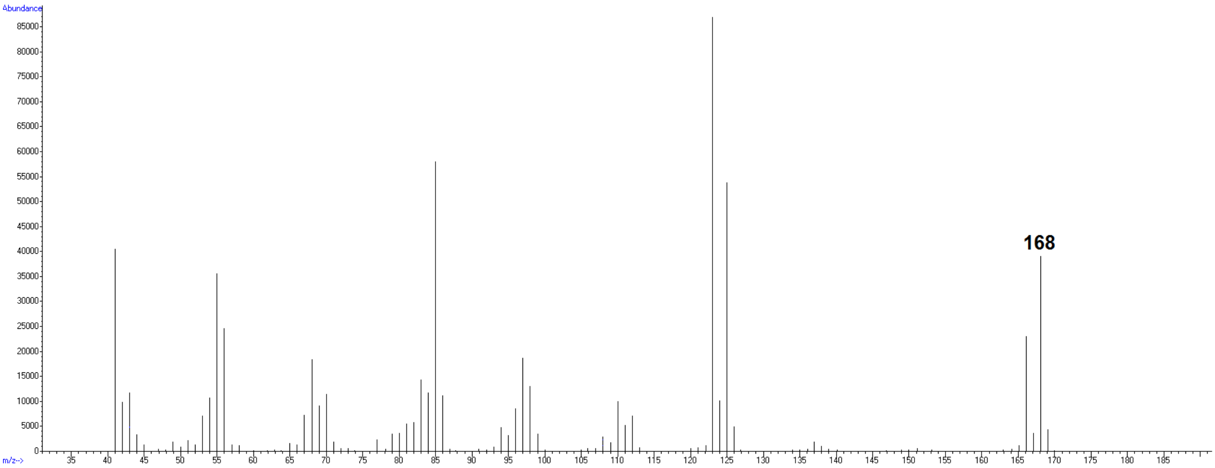


## GC-MS data – biotransformation **32a**

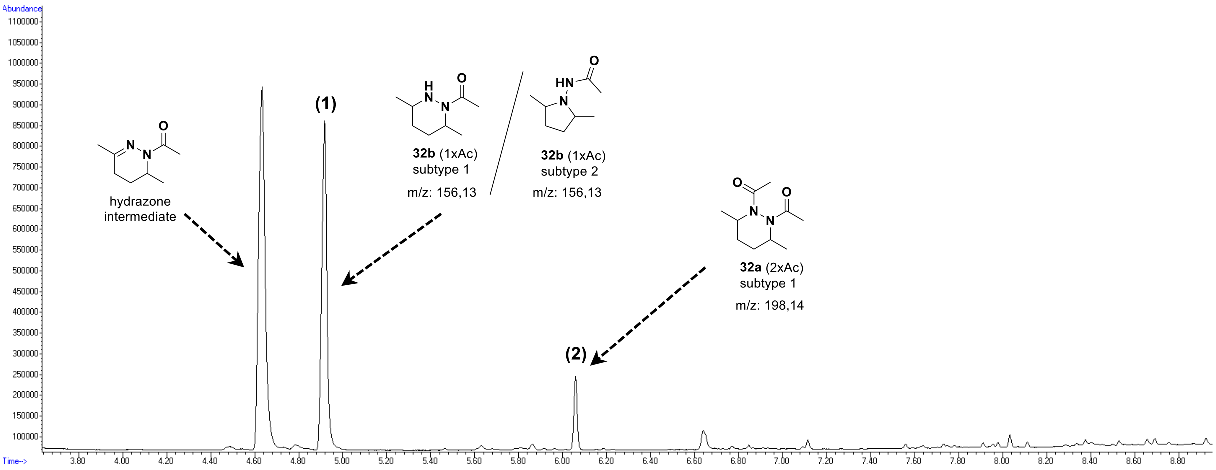

Mass spectrum of **32a** (1):

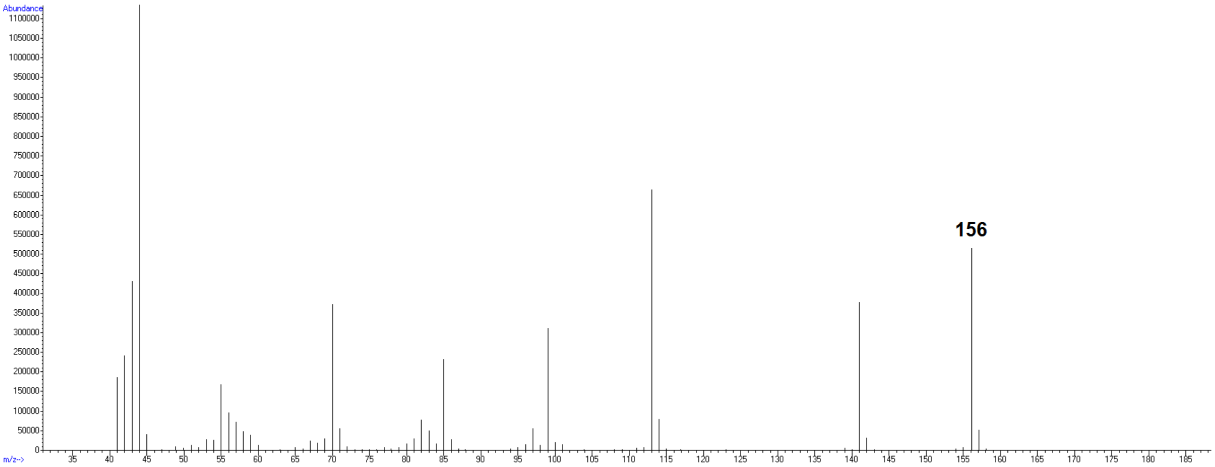


Mass spectrum of **32a** (2):

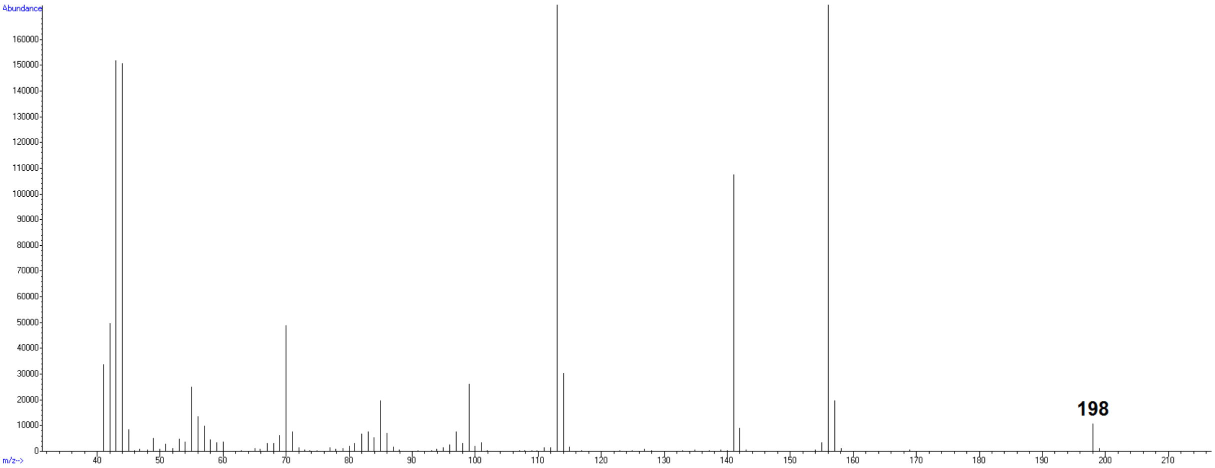


## <u>GC-MS data – biotransformation **32b**</u>

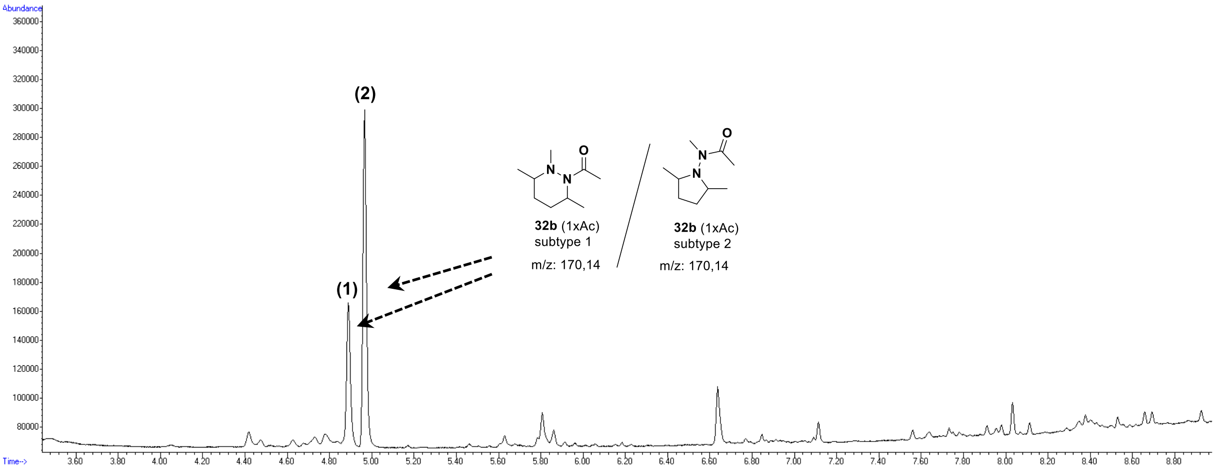

Mass spectrum of **32b** (1):

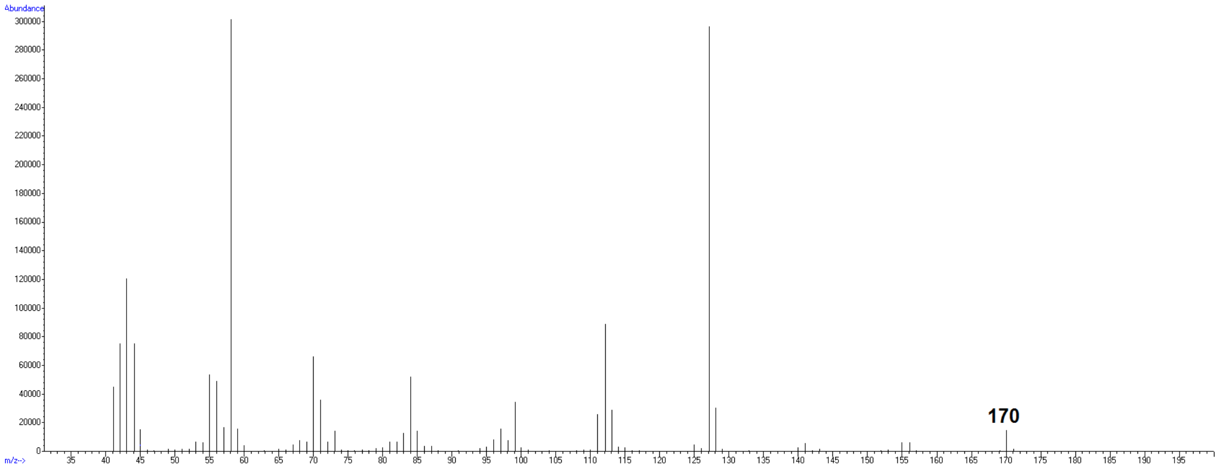


Mass spectrum of **32b** (2):

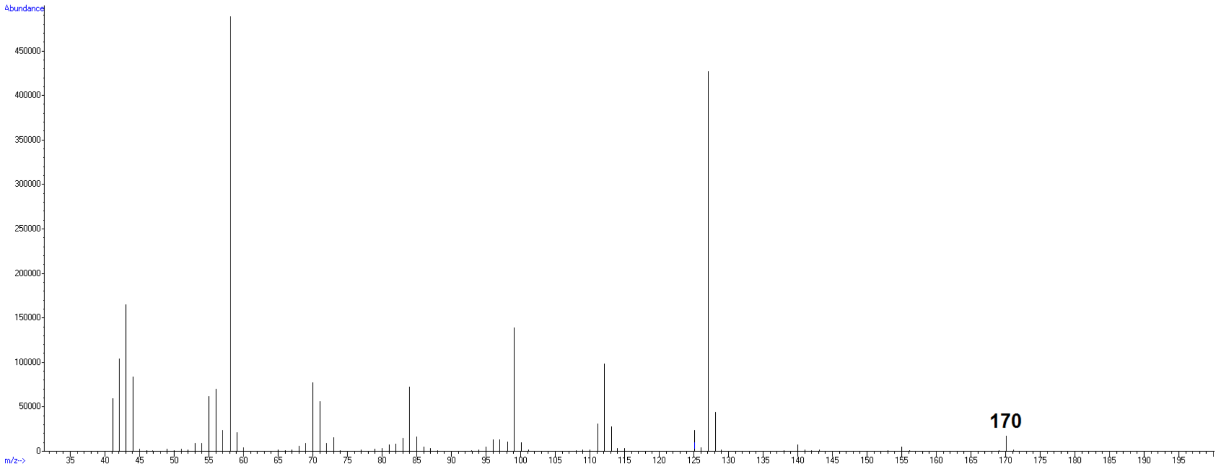


GC-MS data – biotransformation **32c**

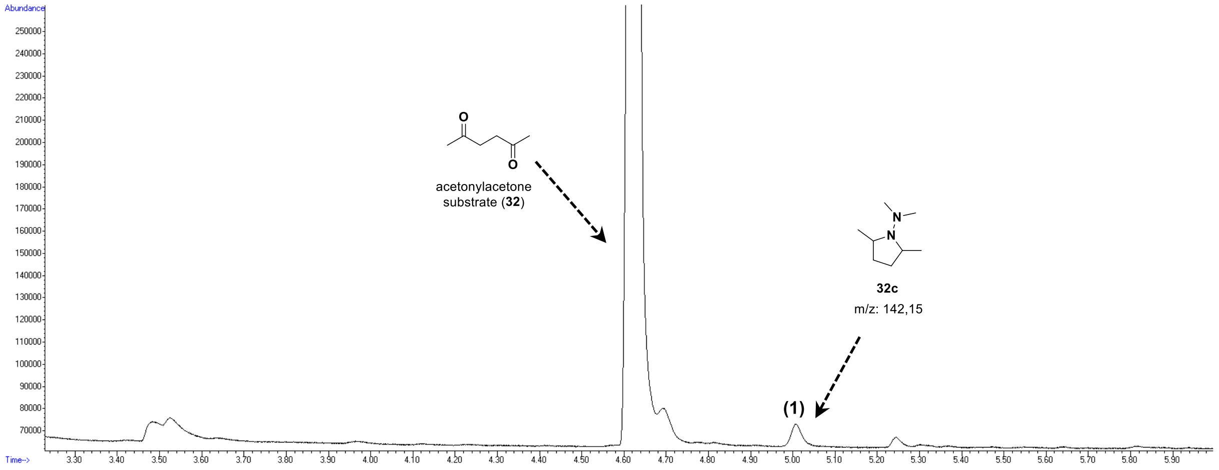

Mass spectrum of **32c** (1):

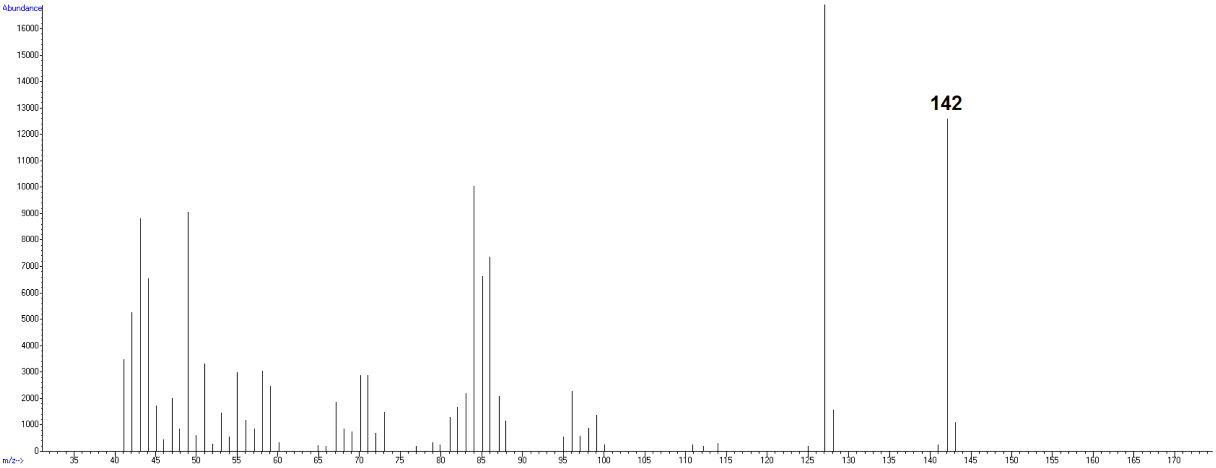


<u>GC-MS data – biotransformation **33a**</u>

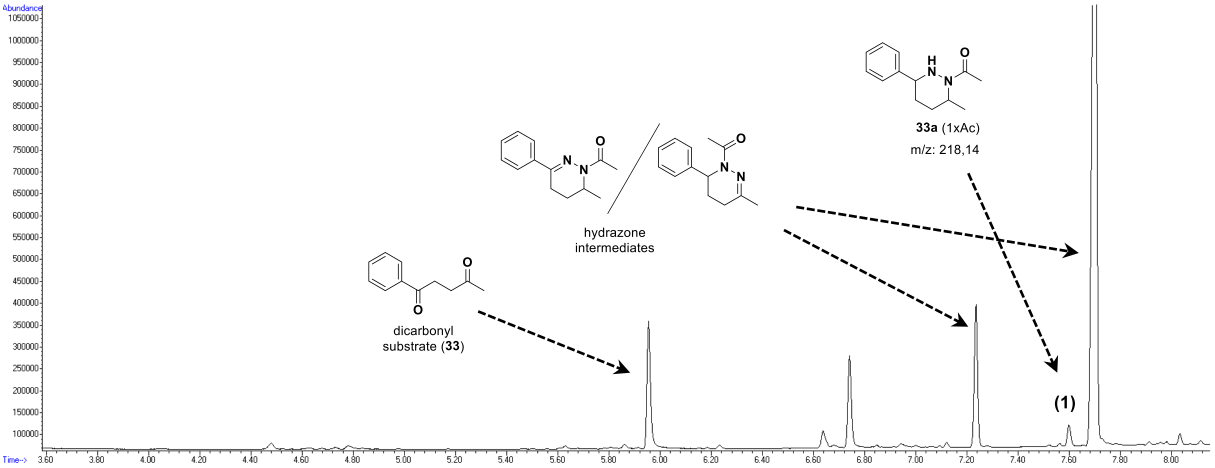


Mass spectrum of **33a** (1):

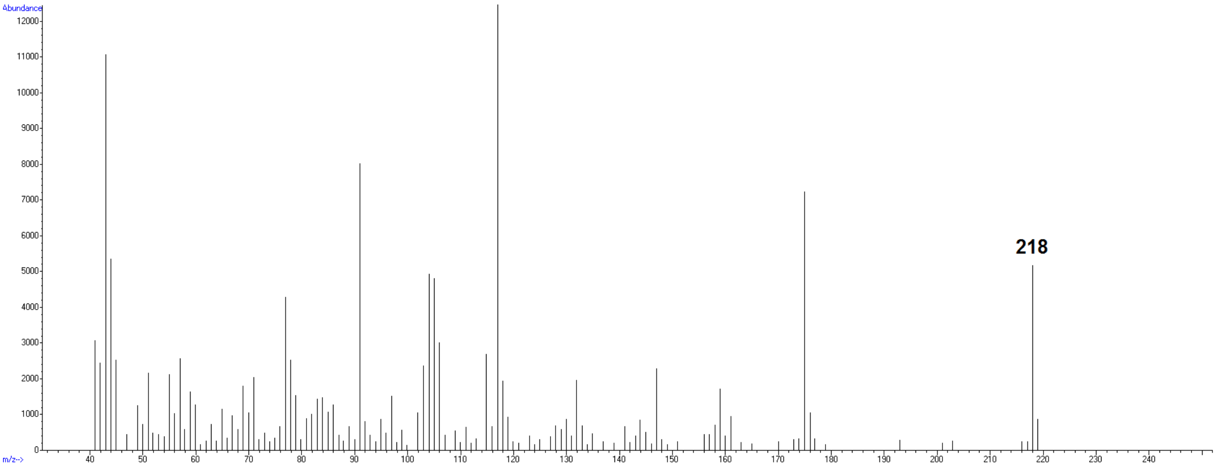

## GC-MS data – biotransformation **34a**

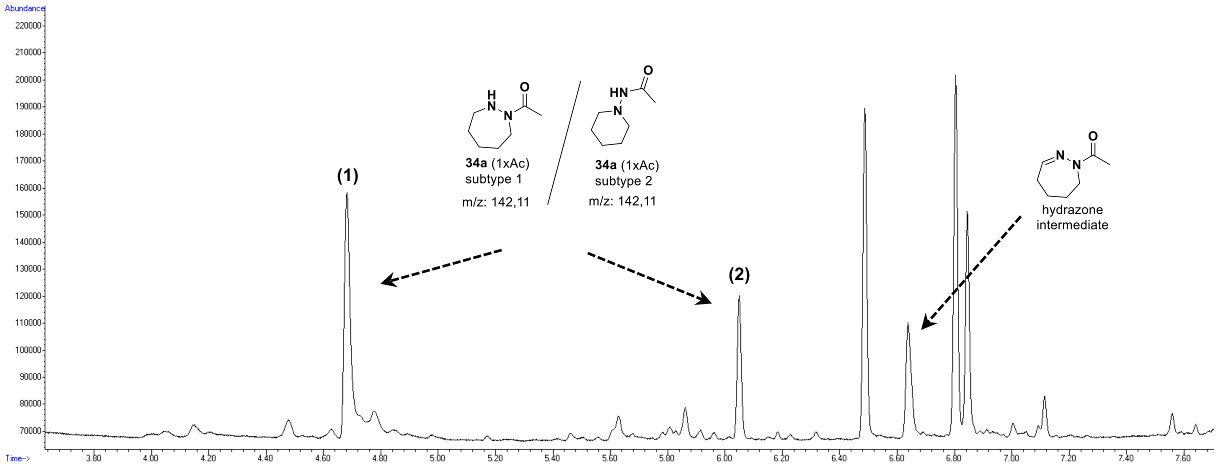


Mass spectrum of **34a** (1):

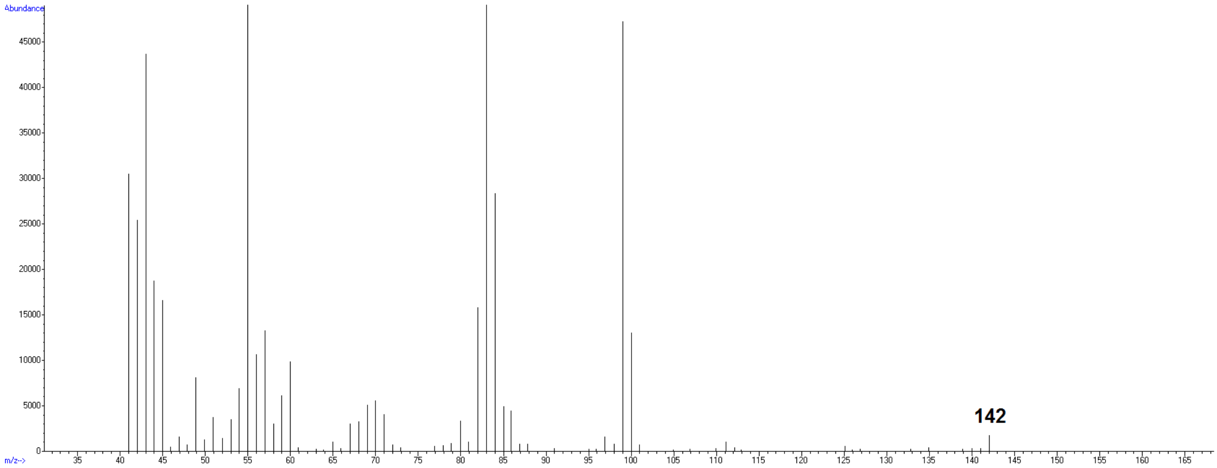


Mass spectrum of **34a** (2):

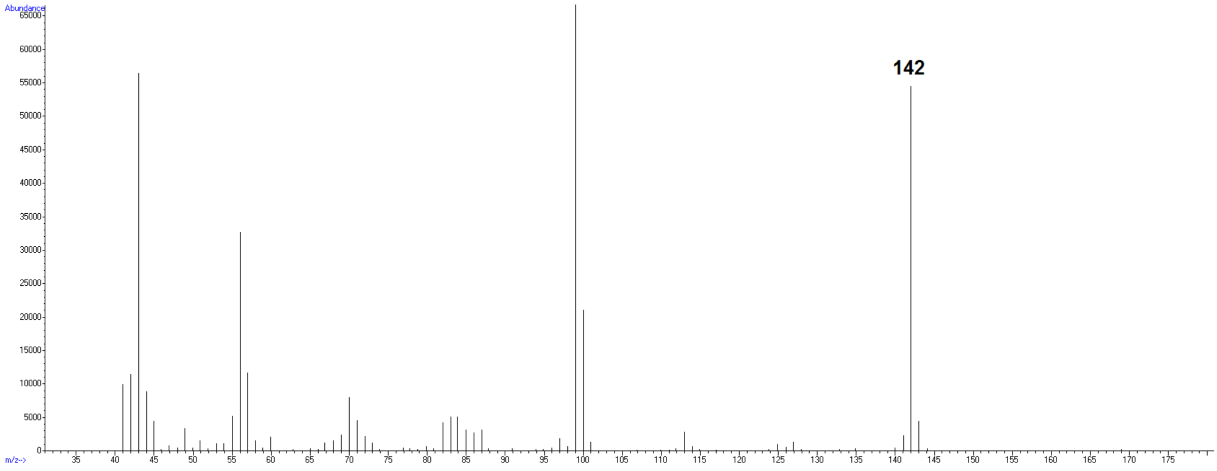

<u>GC-MS data – biotransformation **34b**</u>

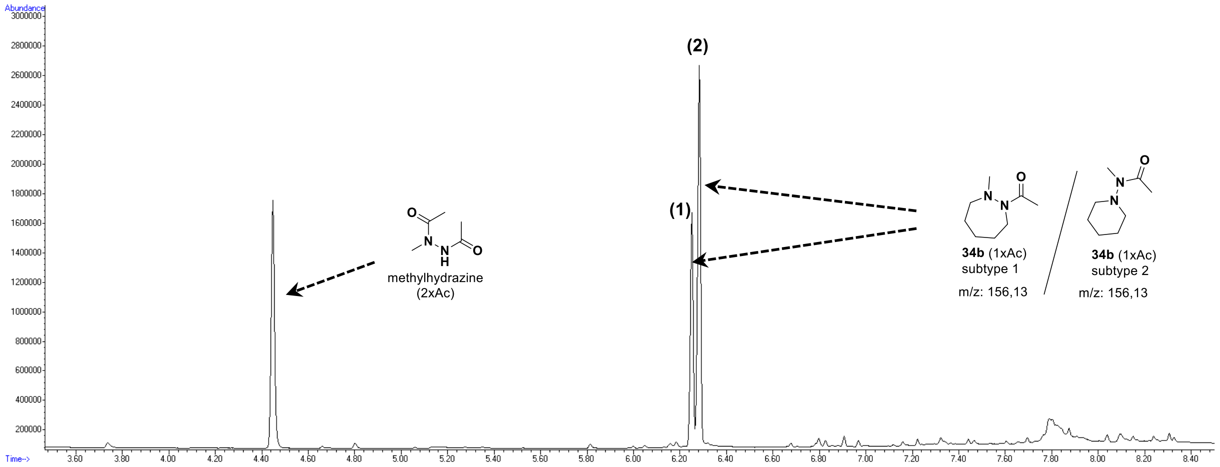


Mass spectrum of **34b** (1):

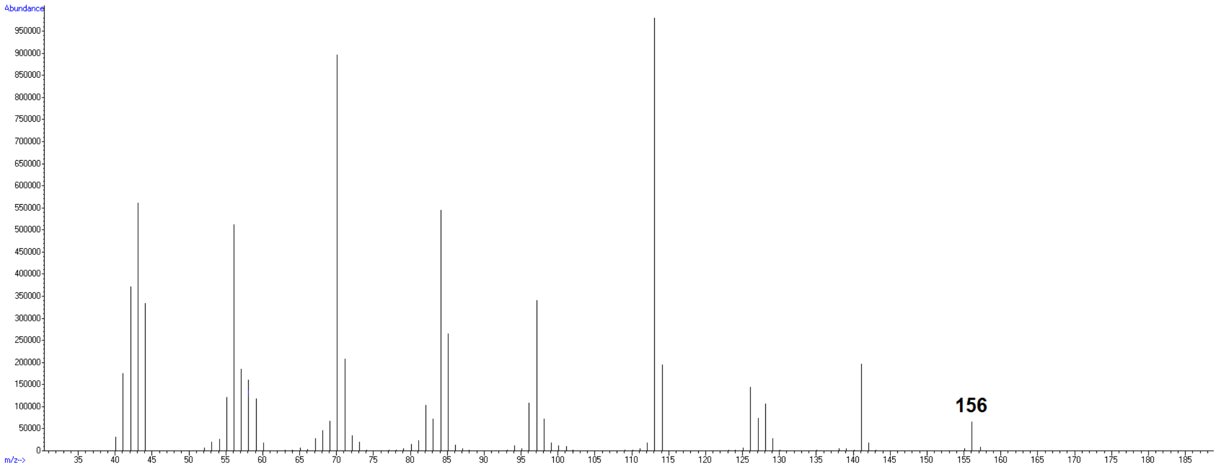


Mass spectrum of **34b** (2):

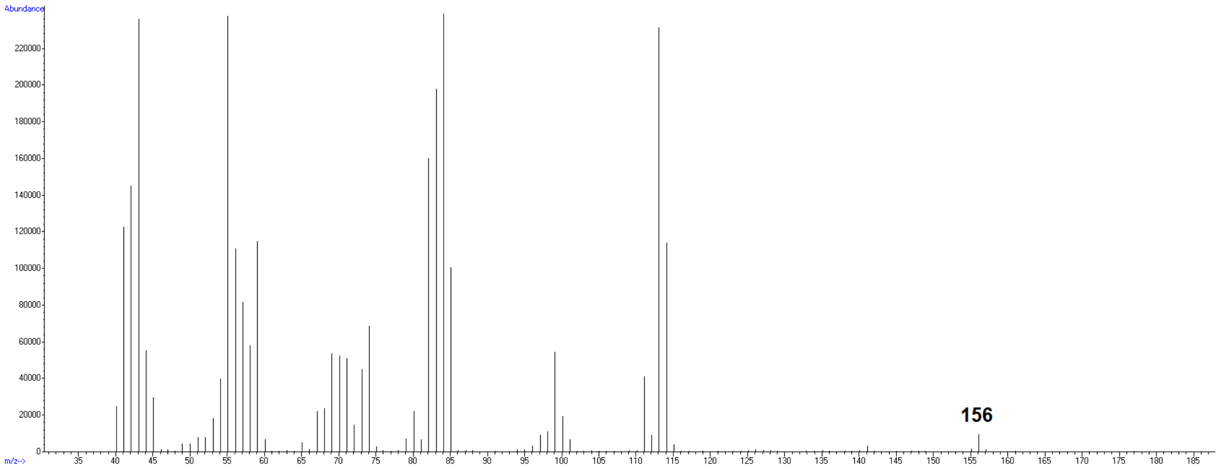

GC-MS data – biotransformation **34c**

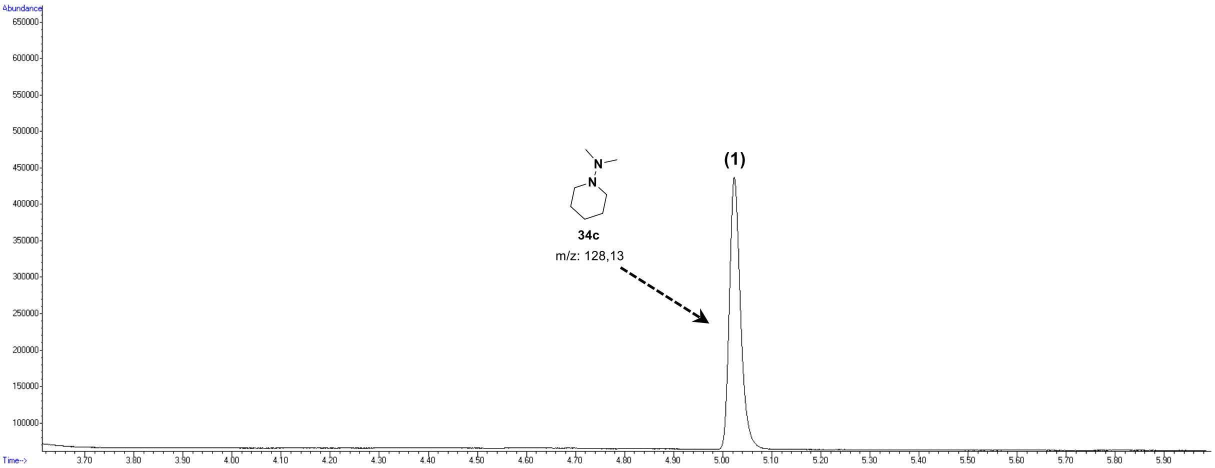


Mass spectrum of **34c** (1):

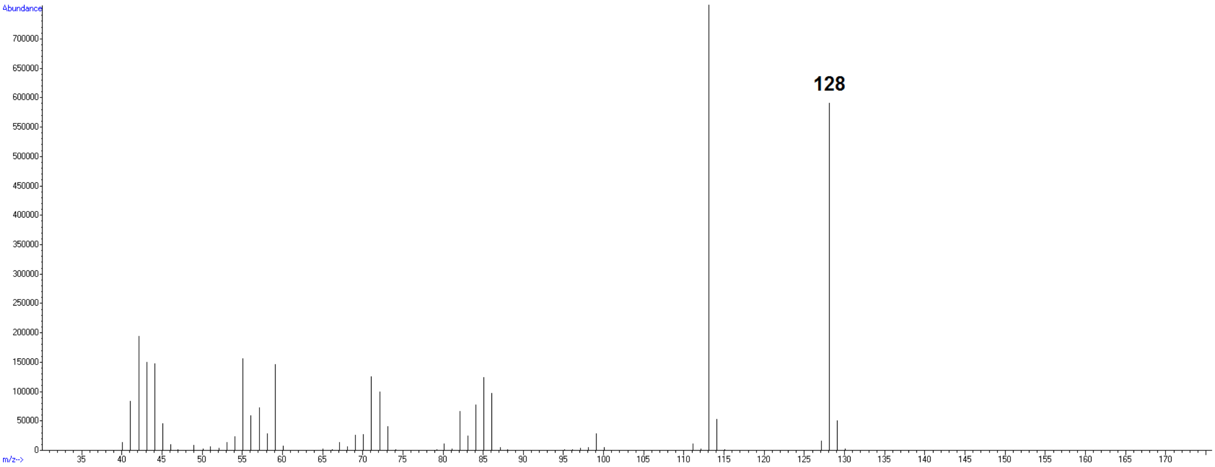


GC-MS data – biotransformation **34f**

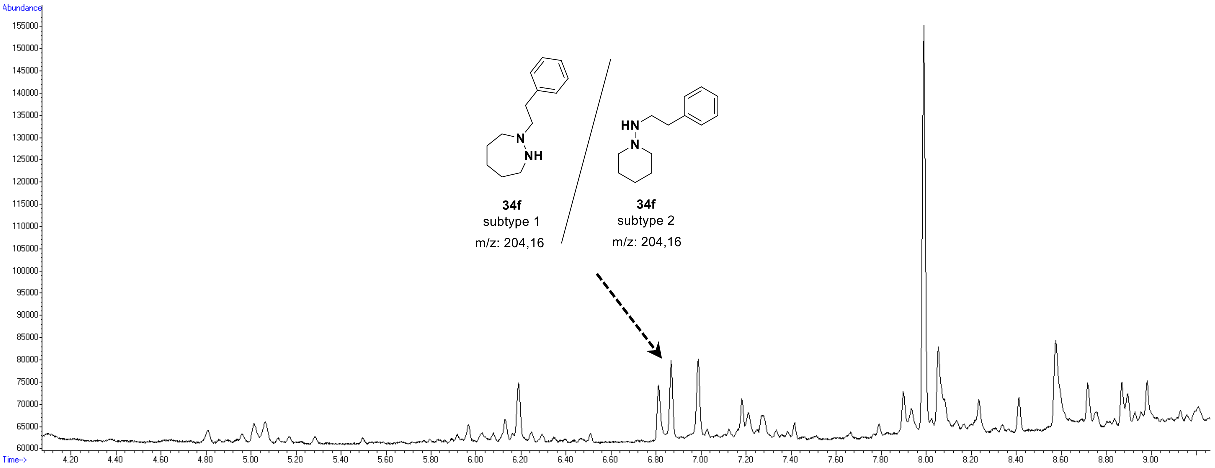

Mass spectrum of **34f** (1):

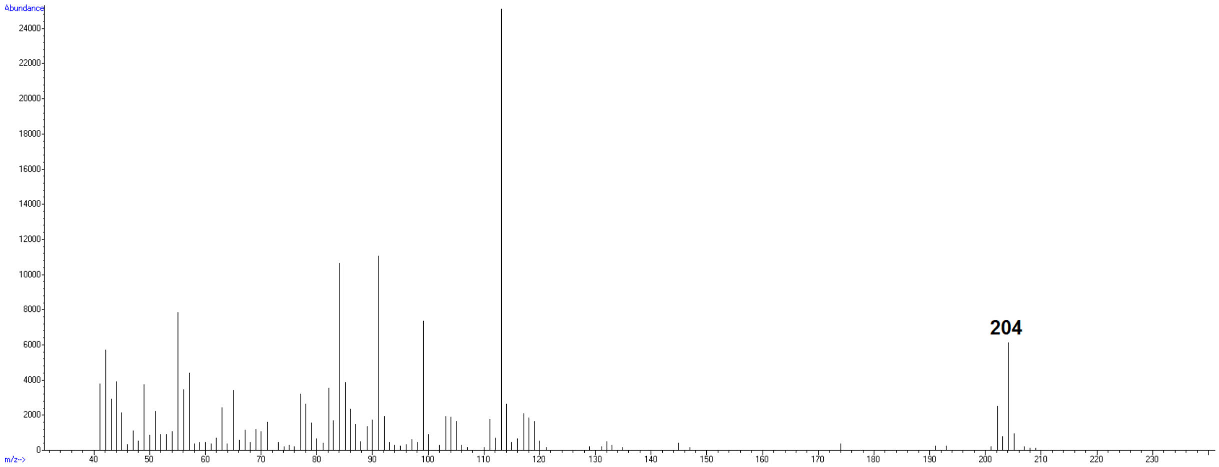


<u>GC-MS data – biotransformation **34g**</u>

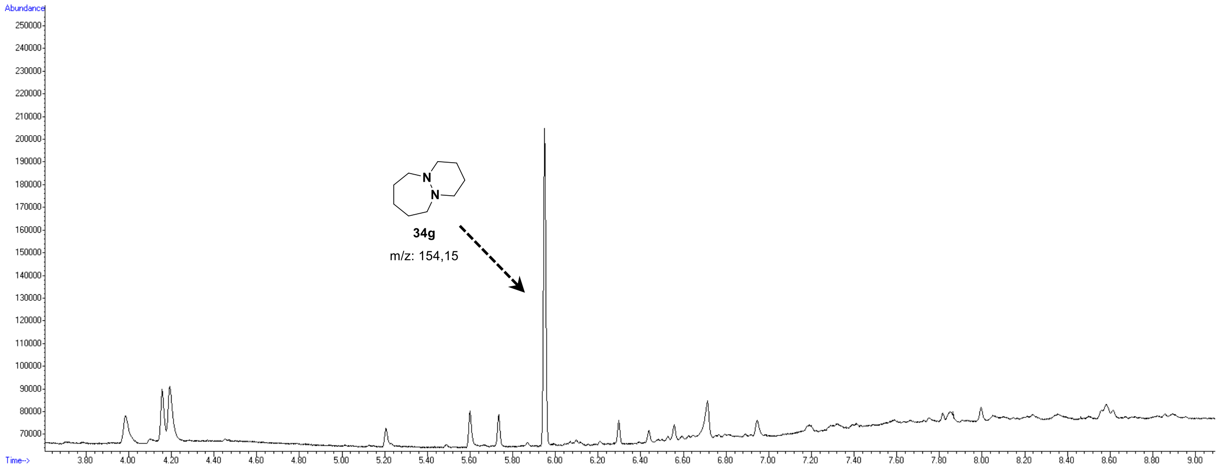


Mass spectrum of **34g** (1):

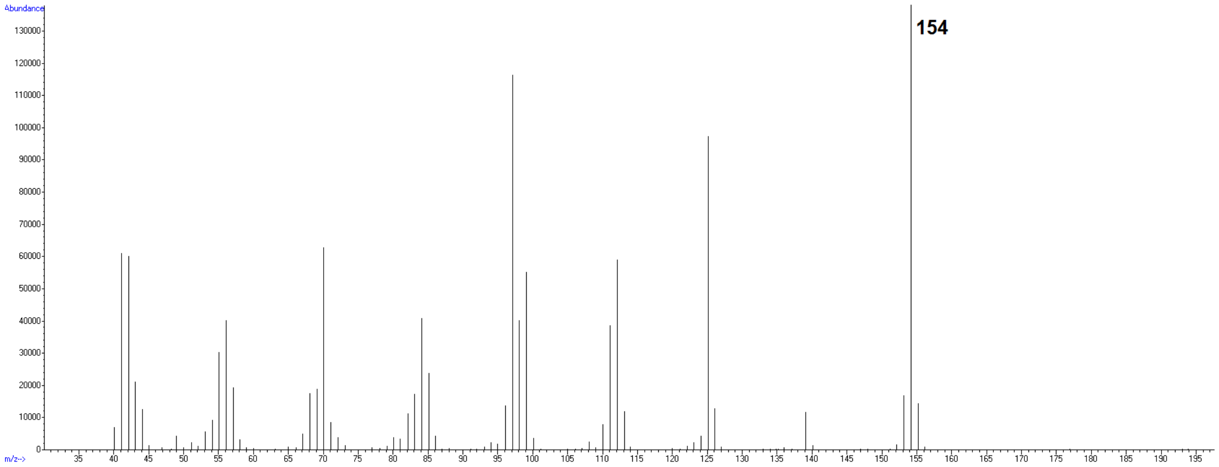

## 5 References


(1) Borlinghaus, N.; Gergel, S.; Nestl, B. M. *ACS Catal.* **2018**, *8* (4), 3727–3732.
(2) Lauterbach, L.; Lenz, O. *J. Am. Chem. Soc.* **2013**, 135, 47, 17897-17905
